# Adherence to unsupervised exercise in sedentary individuals: A randomised feasibility trial of two mobile health interventions

**DOI:** 10.1177/20552076231183552

**Published:** 2023-06-28

**Authors:** Daniel J Bannell, Madeleine France-Ratcliffe, Benjamin James Roy Buckley, Anthony Crozier, Andrew P Davies, Katie L. Hesketh, Helen Jones, Matthew Cocks, Victoria S Sprung, on behalf of the MOTIVATE Team

**Affiliations:** 1 Research Institute for Sport & Exercise Sciences, 4589 Liverpool John Moores University, Liverpool, UK; 2 Liverpool Centre for Cardiovascular Science, 4591 University of Liverpool, Liverpool, UK; 3 Department of Cardiovascular and Metabolic Medicine, Institute of Life Course and Medical Sciences, 4591 University of Liverpool, Liverpool, UK; 4 School of Sport, Exercise and Rehabilitation Sciences, 1724 University of Birmingham, Birmingham, UK

**Keywords:** Exercise, physical activity, feasibility study, behaviour, mHealth, technology

## Abstract

**Introduction:**

Adherence to unsupervised exercise is poor, yet unsupervised exercise interventions are utilised in most healthcare settings. Thus, investigating novel ways to enhance adherence to unsupervised exercise is essential. This study aimed to examine the feasibility of two mobile health (mHealth) technology–supported exercise and physical activity (PA) interventions to increase adherence to unsupervised exercise.

**Methods:**

Eighty-six participants were randomised to online resources (*n *=* *44, females *n *=* *29) or MOTIVATE (*n *=* *42, females *n *=* *28). The online resources group had access to booklets and videos to assist in performing a progressive exercise programme. MOTIVATE participants received exercise counselling sessions supported via mHealth biometrics which allowed instant participant feedback on exercise intensity, and communication with an exercise specialist. Heart rate (HR) monitoring, survey-reported exercise behaviour and accelerometer-derived PA were used to quantify adherence. Remote measurement techniques were used to assess anthropometrics, blood pressure, HbA_1c_ and lipid profiles.

**Results:**

HR–derived adherence rates were 22* *±* *34% and 113* *±* *68% in the online resources and MOTIVATE groups, respectively. Self-reported exercise behaviour demonstrated moderate (Cohen's *d *=* *0.63, CI* *=* *0.27 to 0.99) and large effects (Cohen's *d *=* *0.88, CI* *=* *0.49 to 1.26) in favour of online resources and MOTIVATE groups, respectively. When dropouts were included, 84% of remotely gathered data were available, with dropouts removed data availability was 94%.

**Conclusion:**

Data suggest both interventions have a positive impact on adherence to unsupervised exercise but MOTIVATE enables participants to meet recommended exercise guidelines. Nevertheless, to maximise adherence to unsupervised exercise, future appropriately powered trials should explore the effectiveness of the MOTIVATE intervention.

## Introduction

There is overwhelming evidence that regular physical activity (PA) and exercise are vital for prevention and management of long-term conditions.^1^ Nevertheless, 28% of adults do not meet the global recommendations of 150 min of moderate, or 75 min of vigorous-intensity PA per week.^2^ A contributing factor could be public health strategies, including those within primary healthcare settings, which recommend or employ unsupervised exercise in inactive healthy individuals and those with long-term conditions.^3–6^

Unsupervised exercise has the advantages of low cost and broad accessibility, especially at a population level and when targeted at those with long-term conditions by healthcare professionals. However, meta-analyses suggest unsupervised exercise is ineffective compared to supervised training at improving health outcomes in a variety of long-term conditions.^3–6^ Although several factors may contribute to the ineffectiveness of unsupervised exercise, poor adherence has been cited as an important consideration.^3–6^ Poor adherence (adherence referring specifically to performing the recommended number of exercise sessions) is evident in healthcare settings, such as exercise referral schemes research studies,^7,8^ some of which include the addition of behavioural counselling that is thought to enhance adherence.^9,10^ Poor adherence and compliance (compliance referring specifically to achieving the recommended duration and intensity of exercise sessions) to prescribed exercise are a huge societal problem that impacts on the ability of an intervention to positively change clinical outcomes.^11,12^ Potential reasons for poor adherence and compliance to unsupervised exercise centre around the lack of knowledge, lack of personalisation and lack of support/feedback from exercise specialists.^13,14^ Therefore, effective strategies to overcome these barriers and increase adherence to unsupervised exercise are required.

One potential solution is to explore how technology could be used to enhance personalised exercise prescription and thus improve adherence and compliance to unsupervised exercise. Such technologies, including mobile health (mHealth) wearables, could be used to: (a) provide virtual guidance during exercise (e.g., the use of fitness videos); (b) allow remote exercise support from health professionals that allows communication and feedback (e.g., personalised text messages); and (c) provide instant, individual biometric feedback during exercise (e.g., exercise intensity via heart rate). The primary aim of this study was to, therefore, investigate the feasibility of two mHealth technology supported exercise interventions to increase adherence to unsupervised exercise.

Given that the exercise interventions in the current study are unsupervised and home based/remote, the study also aimed to investigate the feasibility of employing remote measurement techniques, whereby participants collect health outcomes from home, known as a ‘remote clinical trial’. Previous research studies employing unsupervised exercise interventions report high participant dropout^9,10,15^ and missing data for key physiological or clinical outcomes. This may be explained by availability, time and travel distance for research facility visits. Remote physiological data collection may therefore enhance participant retention and minimise data loss in time-intensive studies such as exercise interventions.

The specific objectives were to:

- Determine the feasibility of a home-based exercise and PA counselling intervention, supported by mHealth biometrics;
- Determine the feasibility of a home-based exercise intervention supported by the provision of virtual exercise resources;
- Determine the feasibility and fidelity of measurement techniques to assess adherence to unsupervised home-based exercise interventions;
- Determine the feasibility of collecting basic health outcomes using remote measurement techniques.

## Methods

### Study design

**Figure 1.**
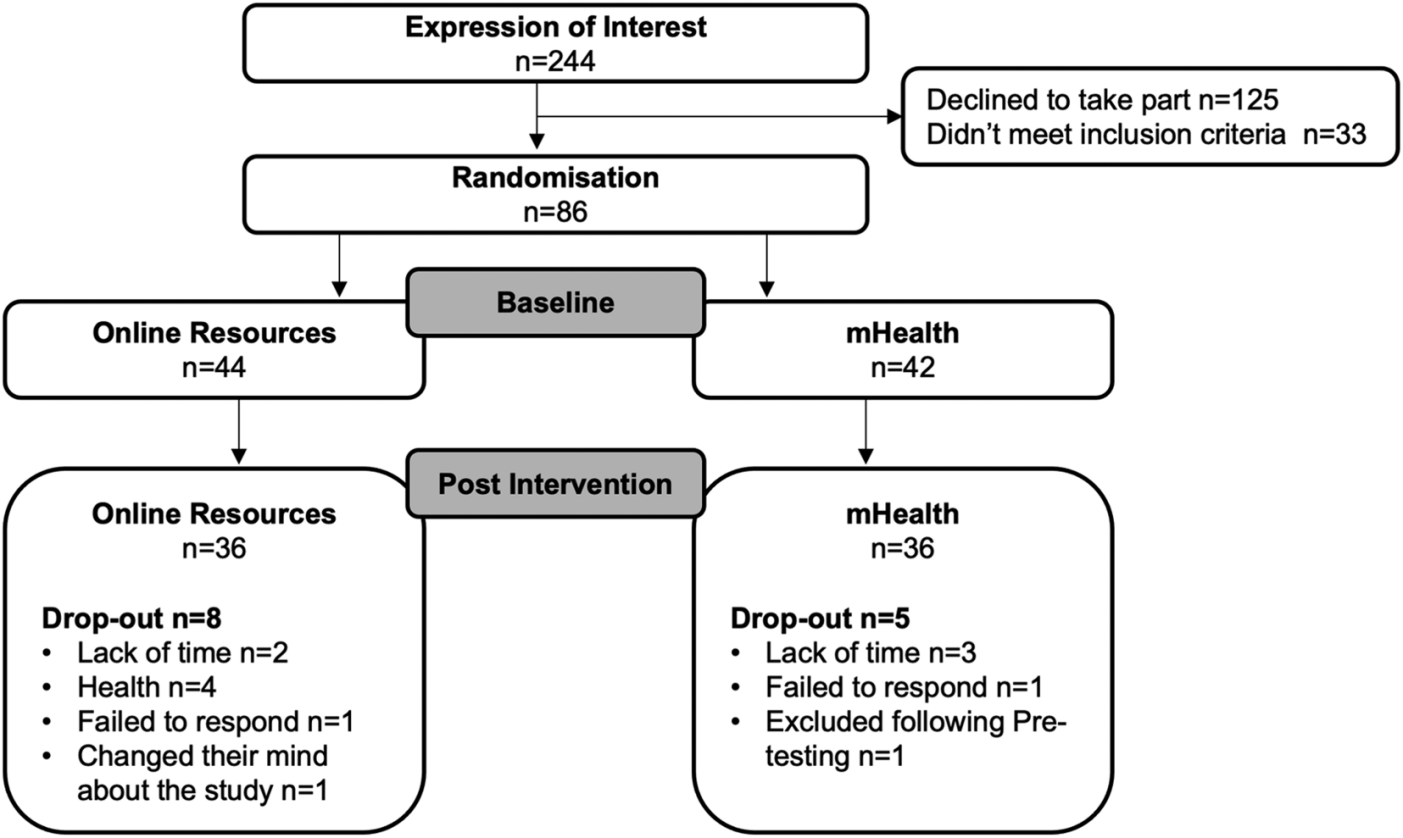
Participant consort diagram.

A feasibility, parallel group randomised trial. Which utilised 1:1 randomisation, whereby participants completed baseline assessments prior to completing a 12-week intervention. Interventions delivered were either home-based exercise supported by online resources (online resources) or mHealth–supported exercise and PA counselling (MOTIVATE), following which measurements were repeated (Figure 1). The reporting of this study conforms with the CONSORT 2010 statement: extension for pilot and feasibility trials^16^ (Supplementary File 1) and TIDieR guidelines for reporting of interventions^17^ (Supplementary File 2). The study was approved by Liverpool John Moores University ethics committee (20/SPS/031) and adhered to the *Declaration of Helsinki*. The protocol was prospectively registered on clinicaltrials.gov (identifier: NCT04979702).

### Participants and setting

Participant recruitment took place between August 2020 and July 2021. Recruitment occurred via emails sent to businesses in the north-west of England and advertisements on social media. Inclusion criteria required that participants were insufficiently active adults (not currently meeting UK PA guidelines^18^), aged between 18 and 75 years, with no known cardiovascular or metabolic disorders and able to exercise safely as deemed by completion of The Physical Activity Readiness Questionnaire (PARQ+; 2020 version). Participants were guided through the PARQ+ by a member of the research team. Exclusion criteria were pregnancy or planning to become pregnant in the next 3 months, <6 months postpartum or breastfeeding* *<* *1 month before recruitment and not owning a smartphone with a data plan or access to Wi-Fi. Participant screening took place via telephone or video call (Zoom Video Communications Inc., 2016). Written informed consent was obtained using the eSignature solution HELLOSIGN, in line with Health Research Authority (HRA) advice (Sept 2018).

### Randomisation and blinding

Participants were randomly allocated using a computer-generated sequence to either the MOTIVATE or online resources groups by an independent researcher. Due to logistical issues with parcels, participants were notified of their group via email prior to baseline testing. By design, the researcher and participants were aware of the group allocation.

### Interventions

All participants undertook a 12-week intervention. Participants could combine exercise training modes to suit individual preference but were encouraged to complete three exercise sessions per week. This frequency is in line with the exercise guidelines for people with type 2 diabetes, which recommends exercise on at least 3 days/week with no more than 2 consecutive days without exercising.^19^ In addition, several studies employing supervised exercise have shown such a frequency to be effective for improving health outcomes.^10,20,21^

#### Online resources

Participants were given access to a website that provided virtual resources and instructions to assist in performing a progressive exercise programme for the 12-week duration. Exercise prescriptions for four different exercise types were available to each participant on the website: moderate-intensity continuous training (MICT), vigorous-intensity training (VIT), high-intensity interval training (HIIT) and resistance training (RT) (details of the exercise prescriptions are outlined in Supplementary Tables 1–4). The website included weekly overviews (in written and video format) of the prescribed sessions for each exercise type, with instructions on total session duration, duration of phases (e.g., warm-up and work and rest periods) and intensity using rate of perceived exertion (RPE, CR-10 scale^22^). Instructional videos with demonstrations were available for individual HIIT and RT sessions.

#### mHealth–supported exercise with PA counselling (MOTIVATE)

The mHealth intervention is based on a recently developed intervention entitled MOTIVATE.^23^ Participants received a series of exercise counselling sessions supported through mHealth technology that consisted of an online coaching platform, a smart phone app and a wrist worn fitness watch. Participants also had access to the website and virtual resources outlined for the online resource group. Participants co-developed a personalised and progressive exercise programme with an exercise specialist (exercise specialists had at an undergraduate degree in sport and exercise science along with specific training in exercise prescription, supervision and motivational interviewing) generally based on the four exercise types outlined above: MICT, VIT, HIIT and RT. Nevertheless, participants could include other activities such as exercise classes, dance or sport depending on personal preference. As part of their exercise counselling sessions, participants were also encouraged to increase their daily habitual PA (PA outside structured planned exercise sessions^24^) by enhancing awareness of the guidelines/recommendations, drawing participant attention to the activity target on their smartwatch (see below) and discussing tips for increasing PA. Built into the 12-week program was behavioural exercise counselling. Participants had four virtual counselling sessions (Zoom Video Communications Inc.) with their exercise specialist (Supplementary Table 5). The content of these meetings was based upon the primary prevention–specific consultation guide developed by the ‘Moving Medicine’ initiative (https://movingmedicine.ac.uk/consultation-guides/condition/adult/primary-prevention/more_minute/) and the Brief Action Planning guide and flow chart developed by The Centre for Collaboration Motivation and Innovation (https://centrecmi.ca/brief-actionplanning/#1502747767988-85883f98-7299).

#### mHealth technology

The exercise prescription was delivered, and progression was monitored by three synchronised mHealth elements:

1. Online platform for exercise specialist (Polar Flow for Coach, www.polar.com/coach): The exercise specialist programmed the agreed co-developed exercise intervention, specifying the agreed number of sessions per week and the type of exercise. The exercise specialist inputted the individual exercise sessions, prescribing the duration and intensity (measured through HR) of each phase within the session, i.e. warm up, workout and cool-down. These detailed exercise sessions were then available as pre-set sessions on the participant's fitness watch. Throughout the intervention, the online platform also provided the exercise specialist with access to the participants training and PA data including daily PA, HR traces from each exercise session, RPE (CR-10 scale ratings^22^) following exercise sessions and individual written comments on exercise sessions provided by the participants;
2. Smart phone app for participant (Polar Flow – Sync & Analyze): Participants used the app to access their exercise programme and to track exercise and PA achievements. All data recorded by the fitness watch were available within the app and participants were able to provide feedback on each exercise session including a session RPE^22^ and written comments on each exercise session;
3. Fitness watch (Polar Ignite, Polar Electro, Finland): Participants were given a Polar Ignite fitness watch, which featured a triaxial accelerometer and optical HR monitor. Progress towards a personalised PA target was displayed throughout the day on the watch screen. Participants were able to access pre-set exercise sessions, designed by their exercise specialist, on the device. The prescribed duration and intensity, via HR zones, were displayed in real time on the watch throughout the exercise session. The watch also provided live visual and haptic (vibration) alerts, coaching participants to execute the session as prescribed (i.e., HR zone and duration). If participants did not want to use pre-set exercise sessions, the monitor screen still provided live visual feedback on exercise time and intensity (current HR).

#### Ongoing virtual communication with exercise specialist

During the first 4 weeks, participants were asked to provide RPE and written comments following all exercise sessions, using the app. The comments related to the appropriateness of the session's duration and intensity and their enjoyment of the session and exercise type. After each recorded exercise session, the exercise specialist used the feedback to send a personalised text message in response to the session. Based on this feedback, the exercise specialist updated the training programme and pre-set exercise sessions as appropriate (changing exercise duration, intensity, rate of progression or type), using the online platform. The aim of this initial period was to refine the exercise sessions to ensure participants had a programme that fit with their current fitness, lifestyle and exercise goals. Following exercise counselling 3 (at 4 weeks), participants received weekly text messages from their exercise specialist. The exercise specialist continued to use the data recorded in the online platform to provide personalised messages, but participants did not have to leave feedback after each exercise session during this period.

The design of the MOTIVATE programme was informed by social cognitive theory.^25^ The intervention components were focussed on Michie and colleagues’ taxonomy of behaviour change technique^26^ categories, including ‘Goals and Planning’, and ‘Feedback and Monitoring’ and motivational interviewing technique processes, including ‘Engaging’ and ‘Evoking’ designed to promote long-term adherence to PA and exercise. These mechanisms were considered appropriate to improve competence to exercise and PA engagement and autonomous motivation for exercise and PA to enhance uptake and maintenance of exercise and PA.

### Outcome measures

#### Intervention feasibility

##### Intervention adherence and compliance

Three methods were used to assess intervention adherence and compliance as a measure of feasibility:

1. *Device-derived measurement of exercise sessions:* Optical HR monitoring was used to record HR during exercise sessions. The online resources group was provided with a Polar OH1 for the duration of the trial and asked to wear it during structured exercise sessions. However, the monitor was not paired with a smart watch or phone, meaning HR data were stored but no real-time/historic feedback was available to participants. As such, the online resources group remained blinded to HR throughout the intervention. For consistency of measurement with the online resources group, data for the MOTIVATE group were collected using a Polar OH1 optical HR monitor paired to the Ignite fitness watch provided as part of the intervention. Using the data uploaded to www.flow.polar.com, the following metrics of adherence and compliance were assessed^10^:
  - Training drop-off: defined as the week during which participants no longer completed any training sessions;
  - Programme Adherence: defined as the % of prescribed sessions completed, where 100% adherence was 36 sessions (3 sessions/wk for 12 wks);
  - Compliance: defined differently for each exercise type (MICT, VIT, HIIT and RT), see Supplementary Table 9 for detailed information but generally refers to the achievement of both a prescribed duration and intensity. Compliance is presented as intention-to-treat and per protocol. Intention-to-treat analysis used all consented participants, and it was assumed that missing HR data represented a missed training session. Per protocol analysis, only data from completed training sessions were included.
2. *Survey-reported exercise behaviour:* Information about participants’ quantity, frequency and intensity of exercise participation was assessed using the Godin Leisure Time Exercise Questionnaire (GLTEQ).^27^ The GLTEQ was administered at baseline, post-intervention and 4, 6 and 8 weeks into the intervention, via an online form (www.googleforms.co.uk). To assess adherence and compliance to the prescribed programmes, the number of sessions of moderate and strenuous intensity exercise of at least 30 min in duration was calculated. This outcome closely reflected the expected prescription during the final week of the intervention;
3. *Device-derived physical activity:* A subset of participants (*n *=* *75; online resources *n *=* *38; MOTIVATE *n *=* *37) wore a wrist-worn tri-axial accelerometer (GT9X, ActiGraph, Pensacola, FL, USA), over a 7-day period at baseline and post-intervention. A recording window of 7 days, starting at midnight on the day of baseline and post-intervention assessments, was utilised. The accelerometer was pre-set to record at 30 Hz to account for transit in the post, while optimising the battery life of the device. Accelerometer data were downloaded using ActiLife version 6.11.9 (ActiGraph, Pensacola, FL, USA) and analysed using the open-source R software package, GGIR (R package for accelerometery) beta v1.6-1 (https://cran.r-project.org/web/packages/GGIR/index.html). GGIR performs autocalibration with the reference of local gravity.^28^ Raw acceleration data were used to compute Euclidean norm minus one (ENMO in mg).^29^ Data were analysed from the first to the final midnight using 5-s epochs. Participants were included in the main analysis if they achieved a minimum of 10 h of wear time for a minimum of 4 days, including at least 1 weekend day. Non-wear was detected if the standard deviation (SD) of two axes was <13 mg with a range of <50 mg in windows of 60 min. The time spent in activity intensities was established using published thresholds.^30,31^ Sedentary time was defined as time accumulated below 45 mg, light PA was defined as ≥46 mg and <100 mg, moderate PA was defined as ≥101 mg and <428 mg and vigorous PA was defined as ≥429 mg. The following metrics of PA were assessed, moderate-to-vigorous PA (MVPA) recorded in ≥10-min bouts (MVPA10+), number of weekly minutes of total, light, moderate, vigorous and MVPA and number of weekly minutes of sedentary time.

##### Qualitative evaluation of intervention feasibility

Semi-structured interviews conducted at post-intervention aimed to understand experiences (barriers, facilitators, actual use of technology and exercise specialist, receptivity to the exercise specialist, perceived appropriateness and suggestions for improvement) with the interventions (technological aspects and exercise prescription and counselling). Two participants from mHealth (*n *=* *1 male) and 4 participants from online resources (*n *=* *2 males) volunteered for post-intervention interviews. All interviews were conducted via telephone or video call (Zoom Video Communications Inc.), according to participant preference, and were structured using a topic guide (Supplementary Tables 6–8).

At post-intervention, all participants were invited to complete an anonymous online survey. The survey was specific to the intervention completed (Supplementary Table 10–11). Questions were developed, piloted within and revised by the research team using appropriate literature.^32^

#### Feasibility of monitoring unsupervised home-based exercise

##### Device-derived measurement of exercise sessions

Participants in the online resources group were asked what proportion of exercise sessions they wore the OH1 HR monitor for 0%, 0–33%, 33–66% and 66–100%.

##### Survey-reported exercise behaviour

The number of moderate and strenuous intensity exercise sessions was assessed by the GLTEQ post-intervention and compared with the number of exercise sessions recorded by the OH1 HR monitor during the final week of training. Comparisons were performed for all participants, MOTIVATE only, online resources only and online resources who reported a wear proportion of 66–100%.

##### Device-derived physical activity

Availability of data using 10 h and 16 h wear-time criteria (minimum of 4 days, including at least 1 weekend day) was assessed.

The ability of the accelerometer to detect exercise sessions was assessed. A subgroup of participants was asked to wear the accelerometer and fitness watch concurrently at week 12 of the intervention. Using the timestamp from PolarFlow, HR and ENMO data were aligned to allow comparison of the proportion of exercise sessions spent in moderate-to-vigorous–intensity activity. Comparisons were performed for MICT, VIT, HIIT and RT sessions. Thresholds used for moderate-to-vigorous activity were ≥101 mg^30^ and ≥60% HR_max_ for accelerometer and HR measures, respectively.

##### Qualitative evaluation of monitoring unsupervised home-based exercise

Semi-structured interviews were conducted at two time points, (1) following baseline and (2) following post-intervention assessments. Five participants (*n *=* *3 males) volunteered for baseline interviews, while two participants from mHealth (*n *=* *1 males) and four participants from online resources (*n *=* *2 males) volunteered for post-intervention interviews. Semi-structured interviews at baseline and post-intervention aimed to learn about experiences (ease and willingness and suggestions for improvement) with all three monitoring approaches.

#### Feasibility of remote measurement techniques

Prior to baseline assessment, participants were mailed, via parcel force, all the necessary assessment resources. Items requiring immediate return (accelerometer and blood sample) were mailed by participants, via Royal Mail to the appropriate destination using pre-paid envelopes, but all other resources were kept by the participant. Prior to post-intervention testing, participants were mailed, via Royal Mail, an accelerometer and blood kit. Written guidelines explaining the assessments were sent alongside testing resources, and videos were available on the study website. Participants received a call from a member of the research team before baseline testing to outline measures and answer any questions. Calls were not routinely provided prior to post-intervention testing. Researchers were remotely available on the day of testing if required. All assessments were completed following an overnight fast, having abstained from caffeine, alcohol and vigorous exercise for 24 h. Data (participant characteristics, anthropometrics and blood pressure) were sent to the research team using an online reporting form (www.googleforms.co.uk).

##### Participant characteristics

Participants completed an online form (www.googleforms.co.uk) to provide basic demographics. This included collection of participant home postcodes which was used to report index of multiple deprivation (IMD).^33^ This index estimates a deprivation score and is categorised into quintiles based on income, employment, education, health, crime, housing and environment information.

##### Anthropometrics

Participants were sent a tape measure (Seca 201, Germany) and electronic scales (Salter, UK). Participants were asked to record their height if known; if not, participants used the tape measure to record their height. Waist circumference was measured in triplicate at the level of the umbilicus. Previous work suggests a strong correlation between self-measured and technician-measured height, body mass^34^ and waist circumference, measured at the umbilicus.^35^

##### Blood pressure

Participants were sent an automated blood pressure monitor, validated by the British and Irish Hypertension Society (UK, Salter BPA-9200-GB; CA, Bios BD215) and measured BP in triplicate following 10-min seated rest. Self-measured blood pressure is a validated approach for monitoring BP, endorsed by the American Heart Association and American Medical Association.^36^

##### Blood sampling

A subset of participants (*n *=* *66; online resources *n *=* *37; MOTIVATE *n *=* *29) took a fasted blood sample which was assessed for glycated haemoglobin concentration (HbA1c) and lipid profile (total cholesterol, HDL cholesterol, LDL cholesterol, non-HDL cholesterol and triglycerides). Participants collected a 500-*µ*l capillary blood sample from a finger prick, using a commercial blood collection kit. Blood collection kit preparation and sample analysis were performed by the Exeter Clinical Laboratory, based at the Royal Devon and Exeter NHS Foundation Trust. Samples were sent directly to the Exeter Clinical Laboratory for analysis. Internal pilot data from the Exeter Clinical Laboratory demonstrate that capillary blood sampling reveals good agreement with standard venous sampling.

##### SF-12 Questionnaire

Participants completed an online version (www.googleforms.co.uk) of the SF-12 Health Survey.

##### Qualitative evaluation of remote measurement techniques

Semi-structured interviews at baseline and-post intervention (*n *=* *9) aimed to learn about experiences (ease and willingness, comprehension and ability to follow instructions, length of time to complete and suggestions for improvement) with remote testing.

Following baseline measures, all participants were invited to complete an anonymous online survey. The survey asked participants about their demographics, experiences of the data collection process and the measures taken (Supplementary Table 12).

#### Sample size

The target sample size that was deemed feasible for this trial was 80 participants (40 per group). Such a sample size is higher than the median value for pilot studies (36 participants per arm) reported in an audit of pilot and feasibility trials registered in the UK clinical research network.^37^ Sample sizes between 24 and 50 have been recommended for feasibility trials.^38^ Our sample size is larger than both of these recommendations.

#### Analytical methods

The potential impact of the interventions on continuous outcomes was reported as mean* *±* *SD and estimates of effect (95% confidence interval) within interventions (baseline to post-intervention). Cohen's *d* effect sizes^39^ were reported and interpreted as small* *<* *0.4, moderate* *=* *0.4–0.8 and large* *>* *0.8.

Spearman's rho coefficient was calculated to determine the association and the bivariate correlation coefficient between GLTEQ and HR monitor–derived and accelerometer and HR monitor–derived outcomes. The (*r*) value was interpreted as 0.0 to 0.2 very weak to negligible correlation, 0.2 to 0.4 weak to low correlation, 0.4 to 0.7 moderate correlation, 0.7 to 0.9 strong, high correlation and 0.9 to 1.0 very strong correlation.^40^ In addition, Bland–Altman analyses determined the level of agreement between GLTEQ and HR monitor–derived and accelerometer and HR monitor–derived outcomes.^41^ Plots with mean difference and limits of agreement (±1.96 SD) and levels of agreement were assessed between the measures.

Data obtained via semi-structured interviews were audio recorded and transcribed verbatim. Interview transcripts were thematically analysed manually via a six-phase process^42,43^: data familiarisation, generating codes, searching for themes, reviewing themes (narrowing in relation to feasibility of remote testing, an mHealth exercise intervention and monitoring unsupervised exercise), defining and naming themes and writing up. Themes were produced based on patterns of response and relevance to the pre-determined categories formed by the research questions. Flexibility in analysis was driven by both the prevalence (number of interviewees articulating the theme) and the importance given to the information. This method of coding allows all interview information to be thematic with focal points for discussion and future recommendations acknowledged.^44^ Primary analysis was conducted by AC with frequent debriefing sessions with the research team to discuss, challenge, and reframe the thematic structure.

The qualitative survey responses from open questions were analysed using a framework approach,^45^ which has been used previously within mixed method studies.^32^

## Results

Between 1 August 2020 and 31 July 2021, *n *=* *244 people expressed an interest in the study; of these, *n *=* *209 were eligible and *n *=* *86 participants were recruited (Figure 1). Participants were randomised to online resources (*n *=* *44, females *n *=* *29) or MOTIVATE (*n *=* *42, females *n *=* *28) groups. Baseline characteristics are presented in Table 1.

**Table 1.** Baseline participant characteristics (mean* *±* *SD).

	MOTIVATE (*n *=* *42)	Online Resources (*n *=* *44)
Age (years)	45* *±* *11	47* *±* *11
Weight (kg)	81.9* *±* *19.4	81.6* *±* *17.0
Height (cm)	168.7* *±* *7.6	167.0* *±* *16.0
BMI (kg/m^2^)	29* *±* *6	31* *±* *16
Waist circumference (cm)	95* *±* *19	97* *±* *14
SBP (mmHg)	115* *±* *15	117* *±* *16
DBP (mmHg)	79* *±* *8	80* *±* *11
MAP (mmHg)	96* *±* *14	98* *±* *13
IMD Quintile	3* *±* *1	3* *±* *1

BMI: body mass index; SBP: systolic blood pressure; DBP: diastolic blood pressure; MA: mean arterial pressure; IMD: index of multiple deprivation.

### Intervention feasibility

#### Intervention adherence and compliance

Adherence and compliance to prescribed exercise, measured via an optical HR monitor (Table 2), indicate participants in the MOTIVATE group exercised more regularly than prescribed. No exercise uptake (completed zero training sessions) was 5% in MOTIVATE (*n *=* *2) and 39% (*n *=* *17) in online resources. During the final week of the intervention, 79% (*n *=* *33) of MOTIVATE participants were completing prescribed exercise sessions compared with 14% (*n *=* *6) in online resources. Supplementary Figure 1 displays the percentage of participants still completing training sessions at each week of the intervention.

**Table 2.** Adherence and compliance assessed by device-derived measurement of exercise sessions.

Outcome	MOTIVATE (*n *=* *42)	Online resources (*n *=* *44)
Number of sessions per week (*n*)	3.4* *±* *2.0	0.7* *±* *1.0
Total adherence (% of 36) (*n*)	113* *±* *68 [*n *=* *41* *±* *24]	22* *±* *34 [*n *=* *8* *±* *12]
Compliance (% of 36 sessions completed correctly)	83* *±* *43	14* *±* *27
Per protocol compliance (% of recorded sessions completed correctly)	73* *±* *24	36* *±* *41

Intention-to-treat data displayed, except for per-protocol analysis for compliance which included only recorded sessions.

The effect size was large for the change in survey-reported exercise behaviour (number of sessions of moderate- and strenuous-intensity exercise obtained from the GLTEQ) from baseline to post-intervention for participants in MOTIVATE (baseline 0.9* *±* *1.1, post-intervention 3.9* *±* *3.1; *d *=* *0.88, *CI *=* *0.49 to 1.26) and moderate in online resources (baseline 1.1* *±* *1.7, post-intervention 3.1* *±* *2.9; *d *=* *0.63, *CI *=* *0.27 to 0.99).

The effect size was moderate for an increase in MVPA10+ for participants in MOTIVATE (58* *±* *125 min; *d *=* *0.47, *CI *=* *−0.06 to 0.98, Table 3). The effect size for a decrease in sedentary time was also moderate for participants in the MOTIVATE group (68* *±* *139; *d *=* *0.49, *CI *=* *−1.0 to 0.04, Table 3).

**Table 3.** Baseline and post-intervention scores for remotely measured outcomes.

Outcome	MOTIVATE					Online resources				
	*N*	Baseline	Post-intervention	95% CI for Baseline to Post Score Difference	Effect Size	*N*	Baseline	Post-intervention	95% CI for baseline to post score difference	Effect Size
*Anthropometrics*										
Weight (kg)	35	81	83	−0.22, 0.45	0.11	35	81	80	−0.74, 0.05	−0.40
BMI (kg.m^2^)	35	28	29	−0.22, 0.45	0.12	35	28	28	−0.72, −0.03	−0.38
Waist circumference (cm)	35	91	89	−0.60, 0.08	−0.26	35	94	89	−0.73, 0.05	−0.39
*Blood pressure*										
SBP (mmHg)	34	114	115	−0.25, 0.42	0.09	35	117	117	−0.32, 0.34	0.01
DBP (mmHg)	35	78	79	−0.30, 0.37	0.04	35	79	79	−0.42, 0.24	−0.09
MAP (mmHg)	35	94	96	−0.13, 0.54	0.21	35	98	98	−0.36, 0.30	−0.03
*Blood samples*										
HbA1c (mmol/mol)	17	38	37	−0.92, 0.08	−0.43	18	36	36	−0.52, 0.40	−0.06
Total Chol (mmol/L)	13	6	5	−0.67, 0.42	−0.13	11	5	5	−0.42, 0.77	0.18
HDL (mmol/L)	13	1	1	−0.55, 0.54	−0.00	12	1	2	−0.34, 0.80	0.24
LDL (mmol/L)	12	3	3	−0.75, 0.39	−0.19	11	3	3	−0.56, 0.62	0.03
Non-HDL (mmol/L)	13	4	4	−0.72, 0.37	−0.18	11	4	4	−0.55, 0.64	0.05
Triglycerides (mmol/L)	11	1	1	−0.51, 0.67	0.08	11	1	1	−0.56, 0.62	0.03
*SF-12*										
Physical (PCS-12)	32	50	52	−0.13, 0.58	0.23	33	52	51	−0.37, 0.32	−0.03
Mental (MCS-12)	32	42	48	0.07, 0.79	0.43	33	43	47	−0.03, 0.67	0.32
*Physical activity*										
MVPA10+	16	81	139	−0.06, 0.98	0.47	21	123	137	−0.32, 0.54	0.12
MVPA	16	472	475	−0.48, 0.50	0.01	21	536	479	−0.69, 0.18	−0.26
Total PA	16	1427	1364	−0.61, 0.37	−0.12	21	1612	1330	−0.81, 0.08	−0.37
Light PA	16	969	897	−0.66, 0.33	−0.17	21	1085	869	−0.76, 0.12	−0.33
Moderate PA	16	459	454	−0.51, 0.47	−0.02	21	518	455	−0.73, 0.14	−0.30
Vigorous PA	16	13	20	−0.26, 0.73	0.24	21	18	24	−0.25, 0.61	0.18
Sedentary time	16	8563	8086	−1.01, 0.04	−0.49	21	8331	8202	−0.52, 0.34	−0.09

SBP: systolic blood pressure; DBP: diastolic blood pressure; MAP: mean arterial pressure; Total Chol: total cholesterol; HDL: high-density lipoprotein cholesterol; LDL: low-density lipoprotein cholesterol; SF-12: 12-Item Short Form Survey; PCS: physical health component score; MCS: mental health component score; PA: physical activity; MVPA: moderate-to-vigorous PA; MVPA10+: MVPA recorded in ≥10-min bouts.

#### Qualitative evaluation of intervention feasibility

Themes that arose from analysis of participants’ interviews are represented in this section using verbatim quotes to depict the participants’ perspective. Three key themes were developed during analysis. These themes included variety and flexibility of the programmes, support mechanisms and barriers to exercise adherence.

#### Theme 1: variety and flexibility of the programmes

Participants in both groups enjoyed the variety of exercise types on offer and the ability to adapt and progress the programme as it was ongoing. However, MOTIVATE participants discussed the importance of their exercise specialist in refining the programme.

‘*… if it was do the same exercises throughout, I think I would have struggled to do the full twelve weeks three, four times a week of HIIT, or it would have felt like a chore.*’ (MLJMU 06, online resources)

‘*I liked the different … varieties of exercise …*’ (MLJMU103, online resources)

‘*I really liked that it had so many different options available to it … I liked that the programme was adaptable, and over time working with [exercise specialist], I was able to tweak it to what I was liking …*’ (MLJMU52, MOTIVATE).

#### Theme 2: support mechanisms

Participants in both groups reported that they felt well supported during the programmes. Online resources participants cited the importance of the resources available and that the website was clear, easy to follow and provided good support to complete the programme:

‘*I found the instructions really clear and the website really useful, and so I intended to use that to guide me each week, so I’d click on one week at a time to see what I should be doing, and initially I found it motivated me to exercise more and get out.*’ (MLJMU103, online resources)

MOTIVATE participants discussed the importance of the relationship built between themselves and the exercise specialist as well as the importance of the text message feedback in this process:

‘*So you kind of develop a bit of a relationship with someone (exercise specialist) who can then work with you. (Exercise specialist) can make suggestions about things that I don't know about, and it did feel very important, yes.*’ (MLJMU 73, MOTIVATE)

‘*… they (text messages) were little jogging memories of what I’d done, and just like little support as well to keep up the good work … the personal angle of it was nice …*’ (MLJMU59, MOTIVATE).

The importance of the support provided through the participant app and fitness watch was also discussed by MOTIVATE participants. Participants particularly liked how the technology allowed them to keep track of their PA and exercise levels and adapt the programme schedule around their lifestyle. The importance of using HR to guide exercise sessions and the greater understanding of exercise intensity through this were also discussed.

‘*I loved seeing the graphs on the (participant app), so I could see which days, like there was essentially a pattern as to what my activity levels were like a week … the graph kind of gave you evidence about what you have been doing.*’ (MLJMU59, MOTIVATE)

‘*… I really like it. I want to keep it … it gives me information about me that I wouldn't otherwise have, so what my heart rate's doing, and it helps me keep track of an exercise*’ (MLJMU 52, MOTIVATE)

However, both groups discussed how the support received could have been improved. MOTIVATE participants cited adding a community aspect to the programme would have been valuable but highlighted that this could have been difficult due to COVID restrictions. MOTIVATE participants also suggested the drop in feedback level (i.e., text messages) across the programme was too drastic and could have been reduced to a lesser extent. Finally, the availability of quick guides for how to use the technology was discussed.

‘*… that community aspect, because I remember finding it really useful having a chat with other participants, and I realise at the moment anything in real life is tricky, but whether there would be a regular opportunity in other times for a monthly meet-up or something so people could actually meet each other and see how they’re doing, I think would be really valuable*’ (MLJMU52, MOTIVATE).

‘*… or at least not drop it (text messages) quite as low as just one text a week*’ (MLJMU52, MOTIVATE).

Online resources participants suggested that the addition of a diary to track their exercise achievements would have been useful alongside prompts to encourage adherence with programmes.

‘*Maybe doing some kind of exercise diary might have helped, you know.*’ (MLJMU 02, online resources)

‘*… maybe a reminder to go and check, like an automatic email to go and check the website more or just a reminder,*’ ‘*Have you done your training session today?*’ (MLJMU 02, online resources)

#### Theme 3: barriers to exercise adherence

Interestingly, the barriers to completing exercise were similar between groups with the external barriers weather, injury and lack of time all discussed, although the adaptability of the home-based interventions was mentioned as a strength here, with participants able to modify sessions when required.

‘*when it was bad weather, I would use the bottom step of our stairs and get a good audio book, and so do it that way instead*’ (MLJMU52, MOTIVATE).

‘*… there's a time issue there that's been more of a struggle …*’ (MLJMU02, online resources).

‘*In fact I actually did more exercise than I probably would have done normally, to the point where I then over-exercised and injured myself, so I had to kind of rein it back in a bit …*’ (MLJMU 103, online resources)

#### Online survey

Sixty-nine (MOTIVATE *n *=* *33, online resources *n *=* *36) participants completed the online survey following the intervention. Based on the survey responses, two key themes and further sub-themes were developed: (a) the COVID-19 pandemic and (b) Motivation, with the sub-themes, (i) accountability, (ii) communication and feedback, (iii) tracking, (iv) flexibility of the intervention and (v) environmental. Supplementary Table 13 shows the frequency of participant positive and negative responses relating to each theme.

### Feasibility of monitoring unsupervised home-based exercise

#### Device-derived measurement of exercise sessions

Thirty-six (82%) participants in the online resources group answered the question what proportion of exercise sessions they wore the OH1 HR monitor. Seventeen (47%) participants wore the monitor for 66–100% of sessions, 2 (6%) wore the monitor 33–66% of sessions, 15 (42%) wore the monitor 1–33% of sessions and 2 (6%) did not wear the monitor for any sessions.

#### Survey-reported exercise behaviour

100% of participants, excluding dropouts, completed the GLTEQ questionnaire at baseline (*n *=* *86) and post-intervention (*n *=* *72). GLTEQs were completed by 73 (100%), 71 (99%) and 65 (90%) participants at 4, 6 and 8 weeks, respectively.

**Figure 2.**
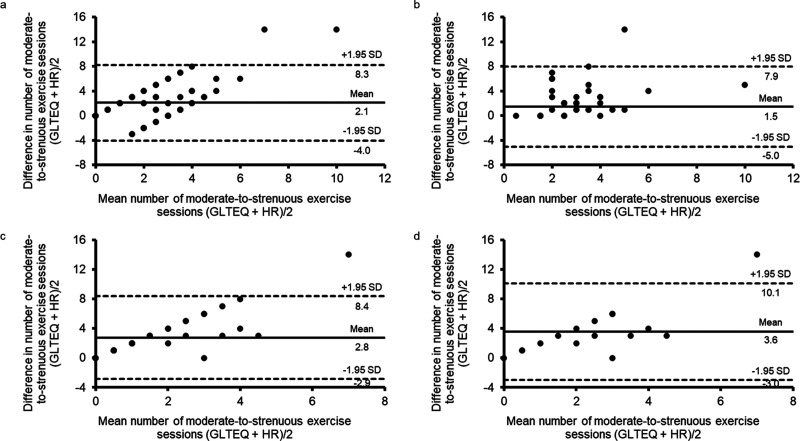
Bland–Altman plots assessing agreement for the number of moderate-to-strenuous exercise sessions recorded by the GLTEQ survey and HR monitor. (a) All participants, (b) MOTIVATE only, (c) online resources only and (d) online resources participants reporting a wear proportion of 66–100%. Solid line shows the mean difference in the number of sessions, and the dashed lines show 95% CI of agreement.

The correlation coefficients comparing the number of moderate- and strenuous-intensity exercise sessions assessed by the GLTEQ and HR monitor were ‘very weak to negligible/weak’ when all participants (*r *=* *0.20), MOTIVATE (*r *=* *−0.13) and online resources (*r *=* *0.35) and online resources participants reporting a wear proportion of 66–100% (*r *=* *0.23) were assessed. Bland–Altman analyses highlighted a mean difference of 2.1* *±* *3.1 sessions, 1.5* *±* *3.3 sessions, 2.8* *±* *2.8 sessions and 3.6* *±* *3.3 sessions for all participants, MOTIVATE only, online resources only and online resources participants reporting a wear proportion of 66–100%, respectively. The limits of agreement for all groups were wide and suggest the presence of a measurement bias for all groups, as the majority of points fall above the zero line (Figure 2).

#### Device-derived physical activity

Accelerometers were sent to 75 participants (MOTIVATE 37; online resources 38) at baseline and post intervention. Accelerometers were returned by 74 (99%) participants at baseline and 72 participants post-intervention (96%). From returned monitors, ≥10 h wear time (≥4 days, including at least 1 weekend day) was achieved by 67 (91%) participants at baseline and 37 (73%) participants post-intervention. When 16 h wear time was used as the criterion, valid wear time dropped to 40 (54%) participants at baseline and 20 (39%) participants post-intervention.

**Figure 3.**
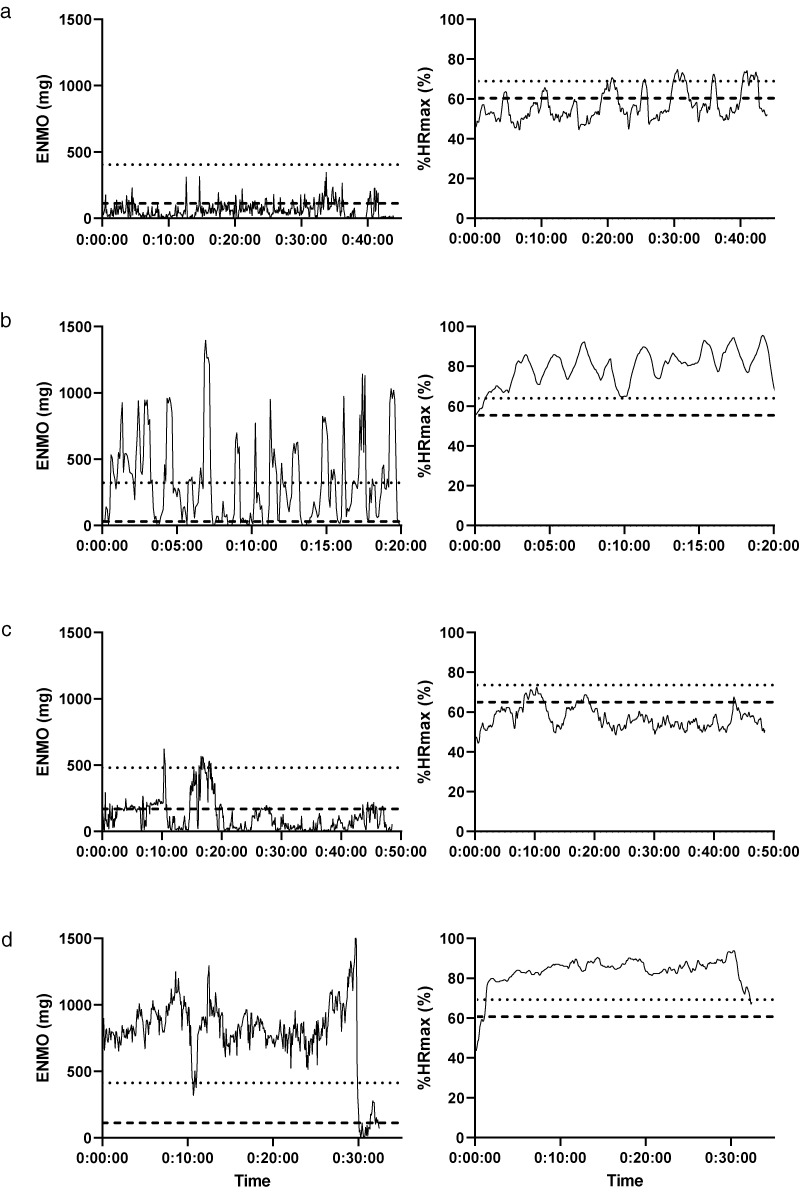
Representative accelerometer (mg) and HR data (%HR_max_) traces recorded throughout bouts of RT (a), HIIT (b), MICT (c) and VIT (d). The dotted line is indicative of the vigorous intensity threshold (>429 mg/70%HR_max_), the dashed line is indicative of the moderate intensity threshold (>101 mg/60%HR_max_).^15^

**Figure 4.**
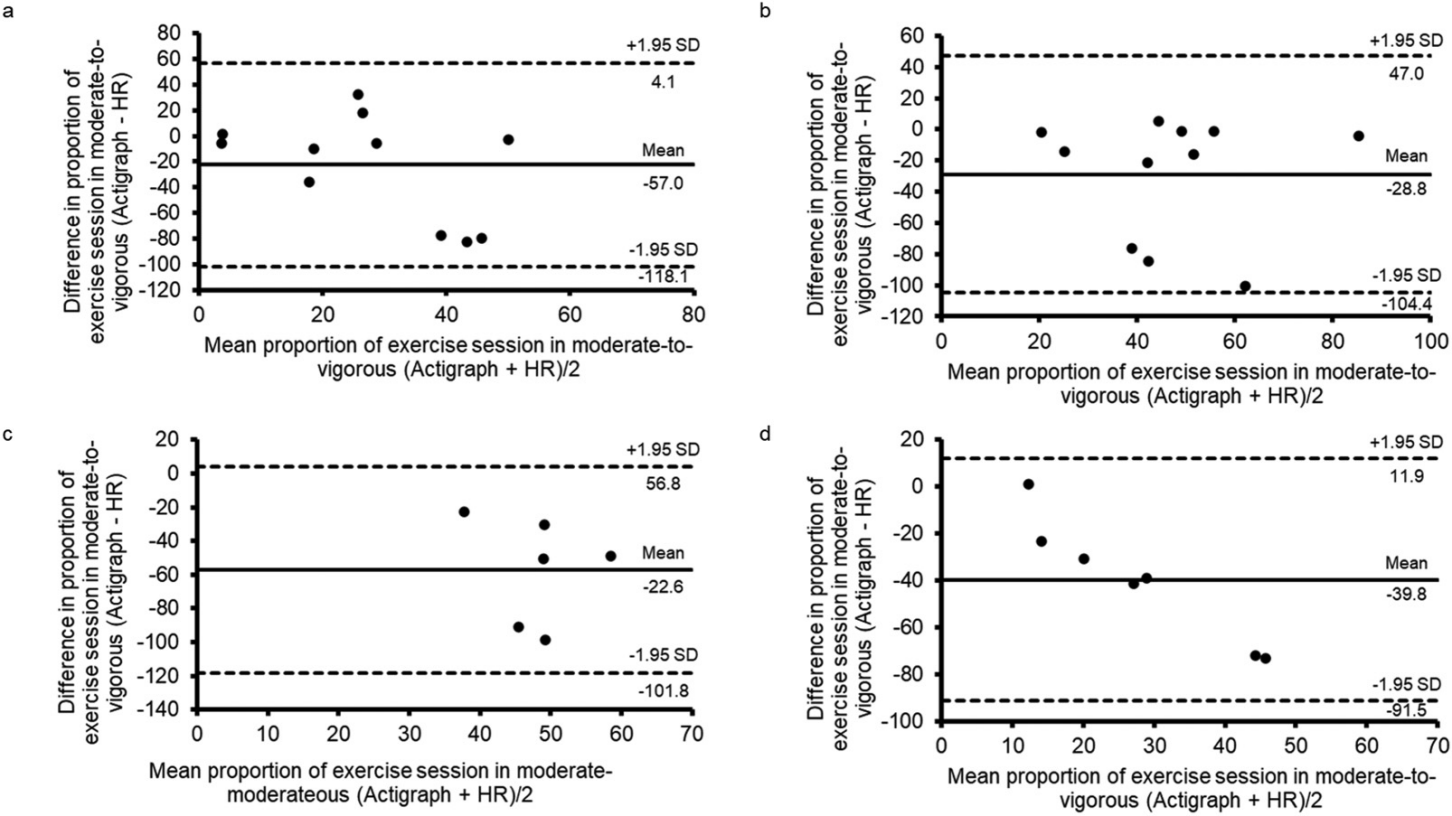
Bland–Altman plots assessing agreement for proportion of exercise session in moderate-to-vigorous–intensity activity recorded by the accelerometer and HR monitor. (a) MICT, (b) VIT, (c) HIIT and (d) RT. Solid line shows the mean difference in % time spent in moderate-to-vigorous–intensity activity, and the dashed lines show 95% CI of agreement.

HR monitor and accelerometer traces were available for 11, 7, 12 and 7 MICT, VIT, HIIT and RT sessions, respectively. Two sessions (1 VIT, 1 HIIT) involved exercise on a cycle ergometer and were excluded from the analysis. Representative accelerometer and HR monitor traces recorded throughout bouts of MICT, VIT, HIIT and RT are shown in Figure 3. Correlation coefficients of the proportion of exercise sessions spent in moderate-to-vigorous intensity activity between the accelerometer and HR monitor were weak, and negative for MICT (*r *=* *−0.16), VIT (*r *=* *−0.15) and HIIT (*r *=* *−0.58) and very weak to negligible for RT (*r *=* *0.14). Bland–Altman analyses show a mean difference of −22.6* *±* *40.5%, −28.8* *±* *38.6%, −57.0* *±* *31.2% and −39.8* *±* *26.4% for MICT, VIT, HIIT and RT, respectively. Limits of agreement for all exercise types were wide and suggest the presence of measurement bias during all exercise types, as the majority of points fall below the zero line (Figure 4). None of the HIIT or RT sessions met the criteria for MVPA10+. Accelerometer and HR monitor traces recorded throughout bouts of VIT and HIIT conducted on a cycle ergometer are shown in Supplementary Figure 2 (these were the only sessions conducted on a cycle ergometer).

#### Qualitative evaluation of monitoring unsupervised home-based exercise

In general participants found the ActiGraph accelerometer acceptable to wear for 7 days: ‘*I forget it's there, to be honest with you … I thought was going to be a much bigger thing than it was …*’ (MLJMU75, MOTIVATE).

Participants in the online resources group struggled with the use of the OH1 HR monitor. Participants reported that it was not very user-friendly and that they often forgot to wear it.

‘*… it would have been more useful to have an activity monitor that was also a heart rate monitor that I just wore for the entirety of the study, because I think there was definitely sometimes where I’d go for a run and realise halfway round that I’d left the heart rate monitor at home, but if had the activity monitor on*’ (MLJMU25, online resources)

### Feasibility of remote measurement techniques

#### Data availability

Participant flow through the study and reasons for dropout are shown in Figure 1. Of the 86 consented participants, 72 (MOTIVATE *n *=* *36; online resources *n *=* *36) completed post-intervention testing, which represented an overall dropout rate of 16% (MOTIVATE 14%; online resources 18%). When dropouts were considered, 84% of data were available. Though with dropouts removed, data availability was 94%. Reasons for missing data are shown in Table 4. Participants lived a range of a 1–284-mile drive (4–303 min) from the university (32* *±* *60 miles; 47* *±* *63 min). The mean IMD quintile was 3* *±* *1.

**Table 4.** Proportion of available data with reasons for remotely measured outcomes at baseline and post-intervention.

	Baseline			Post-intervention		
Outcome	Participants (*n*)	% Data Available (*n*)	Reason for Missing Data (*n*)	Participants (*n*)	% Data Available (*n*)	Reason for Missing Data (*n*)
Anthropometrics	86	100 (86)	N/A	72	97 (70)	Did not want to take (2)
Blood pressure	86	100 (86)	N/A	72	97 (70)	Did not want to take (2)
*Blood samples*
HbA1c	69	97 (67)	Did not want to take (1) Insufficient volume (1)	46	96 (44)	Did not want to take (1) Sample lost in transit (1)
Lipids	69	81 (56)	Did not want to take (1) Insufficient volume (5) Haemolysed (7)	46	72 (33)	Did not want to take (1) Sample lost in transit (1) Insufficient volume (6) Haemolysed (5)
SF-12	86	98 (84)	Did not fill out (1) Completed incorrectly (1)	72	92 (66)	Did not fill out (2) Completed incorrectly (4)

Anthropometrics include height, weight, BMI and waist circumference. Lipid profile includes total cholesterol, high-density lipoprotein cholesterol, low-density lipoprotein cholesterol and triglycerides.

#### Preliminary effectiveness

The effect sizes were moderate for the decreases in body mass (*d *=* *−0.42, CI* *=* *0.76, 0.07) and HbA1c (*d *=* *−0.43, CI* *=* *−0.92, 0.08) in online resources and MOTIVATE groups, respectively (Table 3). There was also a moderate effect–sized improvement in the mental health component of the SF-12 health questionnaire in the MOTIVATE group (*d *=* *0.43, CI 0.07, 0.79).

#### Qualitative evaluation of remote measurement techniques

Four key themes were developed during analysis. These themes included resources given to support testing, understanding of measurements, completing measurements at home and logistics of posting equipment.

#### Theme 1: resources given to support testing

Participants reported that the resources given to help complete the testing (i.e., testing booklet, videos and checklists) were very clear and easy to use and that the visual aspect of the resources were particularly helpful:

‘*Really good and really clear. I liked the visual aspect, looking at the booklet, the photos were there, all really clear. I didn't need to use the videos, but I did look at one of them, just as an additional resource, and again, it was step by step. I knew exactly what I had to do, when I had to do it and how it had to be done … They kind of went hand in hand (booklet & checklist), and yes, they were fab*’ (MLJMU75, MOTIVATE).

Participants also reported that the pre-testing call with a member of the research team was helpful to being able to complete the testing: ‘*… a Zoom call, I thought that was the best resource … I was able to ask the question … just to get total confirmation that I was doing things right*’ (MLJMU10, online resources).

#### Theme 2: understanding of measurements

Participants also reported that they had a good understanding of the measurements, nothing came as a surprise as it was clear what was being measured and why: ‘*I wasn't surprised by anything that was on the list or even where we were discussing what would be involved. I think it was all quite what you’d expect*’ (MLJMU75, MOTIVATE).

#### Theme 3: completing measurements at home

Participants found completing the majority of measurements at home, independently, relatively easy and non-time consuming. They reported that everything was explained well in the pre-testing meeting, and they did not feel the need to have the researcher present as a result: ‘*because everything was explained so well beforehand, I was quite happy just doing it myself … No, no problems …*’ (MLJMU10, online resources group). Conversely, some participants did report that obtaining a finger prick blood sample was noticeably more challenging: ‘*… (blood sample) could have been made easier … it was difficult to see where that mark was … I had to keep kind of checking …*’ (MLJMU75, MOTIVATE).

#### Theme 4: logistics of posting equipment

In general, the logistics of posting testing equipment to participants worked well; participants reported receiving their packages in a timely manner: ‘*They said they were going to send it out on the Thursday, and I received it on the Friday, so it was super-quick*’ (MLJMU75, MOTVATE group) and ‘*I think it came earlier than they’d actually said, and then everything was in there …*’ (MLJMU08, online resources).

#### Online survey

78 (MOTIVATE *n *=* *39, online resources *n *=* *39) participants completed the online survey following baseline measurements. Outcomes from the closed questions are presented in Supplementary Table 14.

## Discussion

The primary aim was to investigate the feasibility of two mHealth technology–supported exercise interventions to increase adherence to unsupervised exercise. The direction and magnitude of the effect sizes suggest that both interventions could be used to invoke potentially meaningful increases in exercise behaviour. Nevertheless, the largest effects were evident following the MOTIVATE intervention, including survey-reported exercise behaviour, time spent in purposeful MVPA (MVPA10+) and adherence to the intervention. We also show that using a combination of methods (i.e., HR monitoring, accelerometery and surveys) may be an essential approach in measuring adherence. Additionally, remote data collection that incorporates self-testing as part of an exercise intervention is feasible. Appropriately powered randomised control trials are now required to explore the effectiveness of the MOTIVATE intervention on enhancing exercise adherence in primary and secondary care.

### Intervention feasibility

#### MOTIVATE

Exercise interventions for insufficiently active individuals, who are recommended unsupervised exercise, need to promote high adherence to impact non-communicable disease. We show adherence of 113* *±* *68% in MOTIVATE, based on sessions captured via the optical HR monitor, demonstrating participants exercised more than the recommended three times per week. Furthermore, compliance in performing the exercise sessions to the correct intensity was 73* *±* *24%, and 79% of participants were still exercising during the final week of the intervention. Findings from our laboratory have previously reported adherence levels to exercise of 30–47% in a UK exercise referral scheme using a similar exercise intervention design to the online resources group in the current study with additional HR monitoring.^10^ Taylor, Doust and Webborn^46^ reported 45% attendance rate (via gym sign-in) to a gym-based UK exercise referral scheme. Moreover, Jung, Locke and Bourne et al.^15^ reported that participants exercised two to four times per week (self-reported) following a brief exercise counselling intervention. Ultimately, the current study provides preliminary evidence that MOTIVATE mediates higher adherence to unsupervised exercise than in previous interventions in insufficiently active individuals. Furthermore, there was evidence of a moderate increase in time spent in purposeful MVPA (MVPA10+; 58* *±* *125 min/week), which suggests an increase in incidental PA of ∼1 h, and a moderate decrease in sedentary behaviour (68* *±* *139 min/week) following MOTIVATE. These changes are broadly in line with previous meta-analyses using technology in self-monitoring interventions.^47,48^ Thus, findings demonstrate that MOTIVATE is helpful in mediating positive changes in structured exercise and habitual PA behaviour.

The post-intervention interviews and survey highlighted participant perceptions of the MOTIVATE intervention. Participants considered the flexibility of the intervention and variety of exercise types as instrumental in facilitating session adherence, which is supported by previous research.^49^ Unsurprisingly, support from the exercise specialist was also important in facilitating adherence. Particularly, participants found the refining of the programme and regular text messages from the exercise specialist significant facilitators. To the authors knowledge, this is the first intervention to use mHealth technology and biometrics to support this two-way connection. We hypothesise that this relationship enhanced participant confidence to perform and manage free-living exercise, which has been deemed essential when initiating new behaviours.^15,50^ Finally, participants highlighted the importance of mHealth technology in facilitating adherence to the intervention, specifically, the role of HR feedback during exercise and the overall PA goal. Previous work has suggested that the availability of live HR data facilitates training programmes by educating participants on how to exercise at an intensity most likely to elicit health changes^51^ and by developing autonomous motivation through feelings of competence.^52^ Given the positive participant perceptions, the current data suggest that MOTIVATE is feasible for implementation within an insufficiently active population.

#### Online resources

Based on sessions captured via the optical HR monitor, participants in the online resources group show exercise adherence levels of 22* *±* *34%, with 14% of participants training during the final week of the intervention. This is lower than MOTIVATE and previous trials using similar intervention design with technology to measure adherence.^10^ However, the post-intervention online survey indicated that many participants did not use the HR monitor to record sessions, suggesting this data likely underrepresents adherence to the online resources intervention. Although the GLTEQ may not reflect adherence to the prescribed exercise programme, the change in the number of sessions of moderate and strenuous intensity exercise increased with a moderate effect size (increase from zero to one exercise session per week). The online resources intervention also had negligible effects on PA and sedentary behaviour. Given the limitations of the accelerometer in detecting MVPA during exercise, this is not surprising, as unlike MOTIVATE, the online resources programme did not have a daily PA target. Participants considered the flexibility and variety of the intervention as important and the resources available on the website as instrumental in facilitating adherence. Taken together, the current study provides evidence on some positive changes in exercise behaviour. Nevertheless, future research studies, that choose to employ the online resources intervention, should consider including behaviour change techniques targeting habitual PA, incorporating self-monitoring and social support based on participant feedback.

#### Feasibility and fidelity of monitoring unsupervised home-based exercise

Monitoring unsupervised home-based exercise presents unique challenges that must be overcome. Data on the dose of exercise and PA are essential to evaluate the efficacy of any intervention.^11^ To the authors knowledge, this is the first study to evaluate the feasibility and fidelity of techniques to measure adherence and compliance to unsupervised exercise programmes. It is important to establish what outcomes each of these measures provide and whether they are sound indicators of adherence. Firstly, optical HR monitoring was used to provide information on the number of planned structured exercise sessions completed and the intensity of those sessions. HR monitoring has previously been advocated as accurate in assessing exercise responses, providing objective personalised data that account for age, body mass and fitness level.^53^ The approach was feasible in MOTIVATE, where participants wore the monitor as part of the intervention and provided rich data. However, the approach was less feasible in the online resources group, where participants used a blinded monitor only for structured exercise, with less than 50% of participants wearing the monitor regularly. Secondly, the GLTEQ was used as a low burden measure of quantity, frequency and intensity of exercise participation,^27^ with the expectation that the survey would closely reflect adherence to the exercise prescription during the final week of the intervention. The survey provided excellent data availability throughout the intervention period (≥90 of data available at all time points). However, the correlation statistics demonstrated poor validity for measuring adherence when compared to the optical HR monitor, and the Bland–Altman analyses indicated inherent measurement error (as suggested by the wide confidence intervals) and the presence of measurement bias. Finally, a wrist-worn accelerometer was used to assess PA. Accelerometer-derived MVPA10+ has been used as the primary outcome measure in PA and exercise interventions where increasing ambulatory activity has been the primary goal.^54^ However, previous reports have cited potential issues when using accelerometers to assess non-ambulatory activity, promoted during the current interventions.^55^ To the authors knowledge, this is the first study to compare HR and accelerometer-defined time in MVPA during structured exercise. The correlation statistics and Bland–Altman analysis demonstrated poor validity for RT and HIIT, both performed through body weight exercises, likely due to the large static component of these complex movements.^55^ However, the correlation statistics and Bland–Altman analysis were also poor for ambulatory MICT and VT. This finding was unexpected and warrants exploration with larger data sets. Therefore, the following are preliminary statements and recommendations for monitoring adherence to unsupervised exercise and PA interventions based on the feasibility data: 1. employ a combination of methods to assess adherence; 2. device-derived HR provides rich data and should be employed, but ways to increase wear time for blinded HR monitors is required, 3. daily PA is important given the potential for compensatory reductions throughout the day,^56^ but accelerometers provide a poor measure of adherence to prescribed exercise and should not be used as standalone measurement, and 4. the GLTEQ survey did not reflect adherence to prescribed exercise, and future research may look at ways of improving validity given the low cost, low burden nature of this tool.

#### Feasibility of remote measurement techniques

Previous research investigating unsupervised exercise interventions reports high amounts of missing data, primarily due to high participant dropout, which jeopardises validity.^9,10,15^ The current study employed a remote clinical trial design with the aim of improving participant retention to outcome measurements. To the authors’ knowledge, this is the first exercise and PA trial to assess the feasibility of such an approach. Importantly, overall participant dropout was lower (16%) than trials employing traditional laboratory testing (24% to 49%).^9,10,15^ A concern with the use of remote clinical trials has been poor protocol adherence, leading to a high proportion of missing data.^57^ However, availability of data for anthropometrics, blood pressure and the SF-12 questionnaire was high (baseline* *≥* *98%; post-intervention* *≥* *92%), with the majority of participants taking a blood sample (baseline 97%; post-intervention 96%). Participant experiences of remote testing also appeared to be positive with interviews highlighting the importance of clear testing resources and support in the process. Although overall data availability was good, the proportion of missing data for the lipid analysis was high, primarily due to issues with sample volume and sample haemolysis. Using participant and research staff experiences, it is suggested that better labelling of tubes with the required volume and more specific instructions prior to assessment could overcome issues. Data suggest that future repeated measures studies should consider employing remote clinical trial designs to reduce participant dropout.

Health inequalities plague research findings due to non-diverse recruitment of participants, specifically those from low-socioeconomic status households.^58,59^ Previous research proposes that a range of variables, other than just occupational income, influence health inequalities.^59^ Furthermore, studies in rural residents suggest that health disparities could be partially due to a lack of access to, knowledge about, and participation in clinical trials.^60,61^ Participants in the current study lived on average 36 (SD: 75) miles drive away from the university. In addition, the observed spread of deprivation scores in both groups (3* *±* *1 AU) highlights the reach of the study design to enhance participation of those typically underrepresented in research. Nevertheless, the current study's recruitment strategies targeted Merseyside employees. Despite this limited targeting, a wide spread of degree and extent of deprivation exists in Merseyside.^62^ Therefore, data are likely representative of the UK population. These preliminary findings highlight the ability to increase access to and inclusivity in clinical trials in those from a range of socioeconomic backgrounds using remote methods.

As expected in the current study's insufficiently active but healthy cohort, changes in health outcomes were negligible. However, data show that a remote clinical trial design for an exercise and PA intervention was feasible and included measures that would be essential in clinical populations. A common measure usually included within exercise and PA intervention studies is cardiorespiratory fitness (CRF). CRF is a strong predictor of all-cause mortality ^63^ and can be improved with exercise. The gold standard measure of CRF is VO_2max_ and is traditionally measured using breath-by-breath oxygen uptake during incremental exercise to exhaustion on a treadmill or cycle ergometer, which is unlikely to be feasible remotely. There are a number of techniques that aim to provide estimates of CRF, with non-exercise prediction models,^64^ step tests^65^ and mHealth technologies^66,67^ showing evidence of accuracy to predict VO_2max_. Although these approaches show promise and ideally should be included in future remote clinical trials, further research is needed to validate their reliability to track changes in CRF and their feasibility for remote testing.

### Limitations and methodological considerations

The decision for two active trial arms rather than a no-treatment control group was informed by the research question, which aimed to inform the design of future unsupervised exercise and PA interventions (RCTs). This strategy allowed us to examine the feasibility of two intervention strategies that differed significantly in their approach, with high levels of participant support versus minimal participant support; thereby adding to the translational value of these data. The authors acknowledge the lack of a follow-up period, and this should be included in the design of future RCTs.

## Conclusion

The current study suggests that both interventions, online resources and MOTIVATE, have a positive impact on exercise behaviour. Yet, greater improvements in adherence and PA were observed within the MOTIVATE intervention arm. Nevertheless, to maximise adherence to unsupervised exercise, future appropriately powered trials should explore the effectiveness of the MOTIVATE intervention in insufficiently active individuals and those with chronic disease. Finally, findings demonstrate the feasibility of remote measurement techniques for data collection in exercise and PA intervention trials.

## Supplemental Material

sj-docx-1-dhj-10.1177_20552076231183552 - Supplemental material for Adherence to unsupervised exercise in sedentary individuals: A randomised feasibility trial of two mobile health interventions Click here for additional data file. Supplemental material, sj-docx-1-dhj-10.1177_20552076231183552 for Adherence to unsupervised exercise in sedentary individuals: A randomised feasibility trial of two mobile health interventions by Daniel J Bannell, Madeleine France-Ratcliffe, Benjamin James Roy Buckley, Anthony Crozier, Andrew P Davies, Katie L. Hesketh, Helen Jones, Matthew Cocks and Victoria S Sprung in DIGITAL HEALTH

sj-docx-2-dhj-10.1177_20552076231183552 - Supplemental material for Adherence to unsupervised exercise in sedentary individuals: A randomised feasibility trial of two mobile health interventions Click here for additional data file. Supplemental material, sj-docx-2-dhj-10.1177_20552076231183552 for Adherence to unsupervised exercise in sedentary individuals: A randomised feasibility trial of two mobile health interventions by Daniel J Bannell, Madeleine France-Ratcliffe, Benjamin James Roy Buckley, Anthony Crozier, Andrew P Davies, Katie L. Hesketh, Helen Jones, Matthew Cocks and Victoria S Sprung in DIGITAL HEALTH

sj-docx-3-dhj-10.1177_20552076231183552 - Supplemental material for Adherence to unsupervised exercise in sedentary individuals: A randomised feasibility trial of two mobile health interventions Click here for additional data file. Supplemental material, sj-docx-3-dhj-10.1177_20552076231183552 for Adherence to unsupervised exercise in sedentary individuals: A randomised feasibility trial of two mobile health interventions by Daniel J Bannell, Madeleine France-Ratcliffe, Benjamin James Roy Buckley, Anthony Crozier, Andrew P Davies, Katie L. Hesketh, Helen Jones, Matthew Cocks and Victoria S Sprung in DIGITAL HEALTH

sj-docx-4-dhj-10.1177_20552076231183552 - Supplemental material for Adherence to unsupervised exercise in sedentary individuals: A randomised feasibility trial of two mobile health interventions Click here for additional data file. Supplemental material, sj-docx-4-dhj-10.1177_20552076231183552 for Adherence to unsupervised exercise in sedentary individuals: A randomised feasibility trial of two mobile health interventions by Daniel J Bannell, Madeleine France-Ratcliffe, Benjamin James Roy Buckley, Anthony Crozier, Andrew P Davies, Katie L. Hesketh, Helen Jones, Matthew Cocks and Victoria S Sprung in DIGITAL HEALTH

sj-docx-5-dhj-10.1177_20552076231183552 - Supplemental material for Adherence to unsupervised exercise in sedentary individuals: A randomised feasibility trial of two mobile health interventions Click here for additional data file. Supplemental material, sj-docx-5-dhj-10.1177_20552076231183552 for Adherence to unsupervised exercise in sedentary individuals: A randomised feasibility trial of two mobile health interventions by Daniel J Bannell, Madeleine France-Ratcliffe, Benjamin James Roy Buckley, Anthony Crozier, Andrew P Davies, Katie L. Hesketh, Helen Jones, Matthew Cocks and Victoria S Sprung in DIGITAL HEALTH

sj-docx-6-dhj-10.1177_20552076231183552 - Supplemental material for Adherence to unsupervised exercise in sedentary individuals: A randomised feasibility trial of two mobile health interventions Click here for additional data file. Supplemental material, sj-docx-6-dhj-10.1177_20552076231183552 for Adherence to unsupervised exercise in sedentary individuals: A randomised feasibility trial of two mobile health interventions by Daniel J Bannell, Madeleine France-Ratcliffe, Benjamin James Roy Buckley, Anthony Crozier, Andrew P Davies, Katie L. Hesketh, Helen Jones, Matthew Cocks and Victoria S Sprung in DIGITAL HEALTH

sj-docx-7-dhj-10.1177_20552076231183552 - Supplemental material for Adherence to unsupervised exercise in sedentary individuals: A randomised feasibility trial of two mobile health interventions Click here for additional data file. Supplemental material, sj-docx-7-dhj-10.1177_20552076231183552 for Adherence to unsupervised exercise in sedentary individuals: A randomised feasibility trial of two mobile health interventions by Daniel J Bannell, Madeleine France-Ratcliffe, Benjamin James Roy Buckley, Anthony Crozier, Andrew P Davies, Katie L. Hesketh, Helen Jones, Matthew Cocks and Victoria S Sprung in DIGITAL HEALTH

sj-docx-8-dhj-10.1177_20552076231183552 - Supplemental material for Adherence to unsupervised exercise in sedentary individuals: A randomised feasibility trial of two mobile health interventions Click here for additional data file. Supplemental material, sj-docx-8-dhj-10.1177_20552076231183552 for Adherence to unsupervised exercise in sedentary individuals: A randomised feasibility trial of two mobile health interventions by Daniel J Bannell, Madeleine France-Ratcliffe, Benjamin James Roy Buckley, Anthony Crozier, Andrew P Davies, Katie L. Hesketh, Helen Jones, Matthew Cocks and Victoria S Sprung in DIGITAL HEALTH

sj-docx-9-dhj-10.1177_20552076231183552 - Supplemental material for Adherence to unsupervised exercise in sedentary individuals: A randomised feasibility trial of two mobile health interventions Click here for additional data file. Supplemental material, sj-docx-9-dhj-10.1177_20552076231183552 for Adherence to unsupervised exercise in sedentary individuals: A randomised feasibility trial of two mobile health interventions by Daniel J Bannell, Madeleine France-Ratcliffe, Benjamin James Roy Buckley, Anthony Crozier, Andrew P Davies, Katie L. Hesketh, Helen Jones, Matthew Cocks and Victoria S Sprung in DIGITAL HEALTH

sj-docx-10-dhj-10.1177_20552076231183552 - Supplemental material for Adherence to unsupervised exercise in sedentary individuals: A randomised feasibility trial of two mobile health interventions Click here for additional data file. Supplemental material, sj-docx-10-dhj-10.1177_20552076231183552 for Adherence to unsupervised exercise in sedentary individuals: A randomised feasibility trial of two mobile health interventions by Daniel J Bannell, Madeleine France-Ratcliffe, Benjamin James Roy Buckley, Anthony Crozier, Andrew P Davies, Katie L. Hesketh, Helen Jones, Matthew Cocks and Victoria S Sprung in DIGITAL HEALTH

sj-docx-11-dhj-10.1177_20552076231183552 - Supplemental material for Adherence to unsupervised exercise in sedentary individuals: A randomised feasibility trial of two mobile health interventions Click here for additional data file. Supplemental material, sj-docx-11-dhj-10.1177_20552076231183552 for Adherence to unsupervised exercise in sedentary individuals: A randomised feasibility trial of two mobile health interventions by Daniel J Bannell, Madeleine France-Ratcliffe, Benjamin James Roy Buckley, Anthony Crozier, Andrew P Davies, Katie L. Hesketh, Helen Jones, Matthew Cocks and Victoria S Sprung in DIGITAL HEALTH

sj-docx-12-dhj-10.1177_20552076231183552 - Supplemental material for Adherence to unsupervised exercise in sedentary individuals: A randomised feasibility trial of two mobile health interventions Click here for additional data file. Supplemental material, sj-docx-12-dhj-10.1177_20552076231183552 for Adherence to unsupervised exercise in sedentary individuals: A randomised feasibility trial of two mobile health interventions by Daniel J Bannell, Madeleine France-Ratcliffe, Benjamin James Roy Buckley, Anthony Crozier, Andrew P Davies, Katie L. Hesketh, Helen Jones, Matthew Cocks and Victoria S Sprung in DIGITAL HEALTH

sj-docx-13-dhj-10.1177_20552076231183552 - Supplemental material for Adherence to unsupervised exercise in sedentary individuals: A randomised feasibility trial of two mobile health interventions Click here for additional data file. Supplemental material, sj-docx-13-dhj-10.1177_20552076231183552 for Adherence to unsupervised exercise in sedentary individuals: A randomised feasibility trial of two mobile health interventions by Daniel J Bannell, Madeleine France-Ratcliffe, Benjamin James Roy Buckley, Anthony Crozier, Andrew P Davies, Katie L. Hesketh, Helen Jones, Matthew Cocks and Victoria S Sprung in DIGITAL HEALTH

sj-docx-14-dhj-10.1177_20552076231183552 - Supplemental material for Adherence to unsupervised exercise in sedentary individuals: A randomised feasibility trial of two mobile health interventions Click here for additional data file. Supplemental material, sj-docx-14-dhj-10.1177_20552076231183552 for Adherence to unsupervised exercise in sedentary individuals: A randomised feasibility trial of two mobile health interventions by Daniel J Bannell, Madeleine France-Ratcliffe, Benjamin James Roy Buckley, Anthony Crozier, Andrew P Davies, Katie L. Hesketh, Helen Jones, Matthew Cocks and Victoria S Sprung in DIGITAL HEALTH

sj-docx-15-dhj-10.1177_20552076231183552 - Supplemental material for Adherence to unsupervised exercise in sedentary individuals: A randomised feasibility trial of two mobile health interventions Click here for additional data file. Supplemental material, sj-docx-15-dhj-10.1177_20552076231183552 for Adherence to unsupervised exercise in sedentary individuals: A randomised feasibility trial of two mobile health interventions by Daniel J Bannell, Madeleine France-Ratcliffe, Benjamin James Roy Buckley, Anthony Crozier, Andrew P Davies, Katie L. Hesketh, Helen Jones, Matthew Cocks and Victoria S Sprung in DIGITAL HEALTH

sj-eps-16-dhj-10.1177_20552076231183552 - Supplemental material for Adherence to unsupervised exercise in sedentary individuals: A randomised feasibility trial of two mobile health interventions Click here for additional data file. Supplemental material, sj-eps-16-dhj-10.1177_20552076231183552 for Adherence to unsupervised exercise in sedentary individuals: A randomised feasibility trial of two mobile health interventions by Daniel J Bannell, Madeleine France-Ratcliffe, Benjamin James Roy Buckley, Anthony Crozier, Andrew P Davies, Katie L. Hesketh, Helen Jones, Matthew Cocks and Victoria S Sprung in DIGITAL HEALTH

sj-eps-17-dhj-10.1177_20552076231183552 - Supplemental material for Adherence to unsupervised exercise in sedentary individuals: A randomised feasibility trial of two mobile health interventions Click here for additional data file. Supplemental material, sj-eps-17-dhj-10.1177_20552076231183552 for Adherence to unsupervised exercise in sedentary individuals: A randomised feasibility trial of two mobile health interventions by Daniel J Bannell, Madeleine France-Ratcliffe, Benjamin James Roy Buckley, Anthony Crozier, Andrew P Davies, Katie L. Hesketh, Helen Jones, Matthew Cocks and Victoria S Sprung in DIGITAL HEALTH

sj-doc-18-dhj-10.1177_20552076231183552 - Supplemental material for Adherence to unsupervised exercise in sedentary individuals: A randomised feasibility trial of two mobile health interventions Click here for additional data file. Supplemental material, sj-doc-18-dhj-10.1177_20552076231183552 for Adherence to unsupervised exercise in sedentary individuals: A randomised feasibility trial of two mobile health interventions by Daniel J Bannell, Madeleine France-Ratcliffe, Benjamin James Roy Buckley, Anthony Crozier, Andrew P Davies, Katie L. Hesketh, Helen Jones, Matthew Cocks and Victoria S Sprung in DIGITAL HEALTH

sj-docx-19-dhj-10.1177_20552076231183552 - Supplemental material for Adherence to unsupervised exercise in sedentary individuals: A randomised feasibility trial of two mobile health interventions Click here for additional data file. Supplemental material, sj-docx-19-dhj-10.1177_20552076231183552 for Adherence to unsupervised exercise in sedentary individuals: A randomised feasibility trial of two mobile health interventions by Daniel J Bannell, Madeleine France-Ratcliffe, Benjamin James Roy Buckley, Anthony Crozier, Andrew P Davies, Katie L. Hesketh, Helen Jones, Matthew Cocks and Victoria S Sprung in DIGITAL HEALTH

## References

[1] Pedersen BK Saltin B . Exercise as medicine – evidence for prescribing exercise as therapy in 26 different chronic diseases. Scand J Med Sci Sports 2015; 25: 1–72. 10.1111/sms.12581 26606383

[2] Guthold R Stevens GA Riley LM , et al. Worldwide trends in insufficient physical activity from 2001 to 2016: a pooled analysis of 358 population-based surveys with 1·9 million participants. The Lancet Global Health 2018; 6: e1077–e1086. 30193830 10.1016/S2214-109X(18)30357-7

[3] Umpierre D Ribeiro PAB Kramer CK , et al. Physical activity advice only or structured exercise training and association with HbA1c levels in type 2 diabetes: a systematic review and meta-analysis. JAMA 2011; 305: 1790–1799. 21540423 10.1001/jama.2011.576

[4] Vemulapalli S Dolor RJ Hasselblad V , et al. Supervised vs unsupervised exercise for intermittent claudication: a systematic review and meta-analysis. Am Heart J 2015; 169: 924–937. e923. 26027632 10.1016/j.ahj.2015.03.009

[5] Lacroix A Hortobagyi T Beurskens R , et al. Effects of supervised vs. unsupervised training programs on balance and muscle strength in older adults: a systematic review and meta-analysis. Sports Med 2017; 47: 2341–2361. 28573401 10.1007/s40279-017-0747-6

[6] Fennell C Peroutky K Glickman E . Effects of supervised training compared to unsupervised training on physical activity, muscular endurance, and cardiovascular parameters. MOJ Orthop Rheumatol 2016; 5: 00184.

[7] Morgan O . Approaches to increase physical activity: reviewing the evidence for exercise-referral schemes. Public Health 2005; 119: 361–370. 15780323 10.1016/j.puhe.2004.06.008

[8] Pavey T Taylor A Hillsdon M , et al. Levels and predictors of exercise referral scheme uptake and adherence: a systematic review. J Epidemiol Community Health 2012; 66: 37. 22493474 10.1136/jech-2011-200354

[9] Roy M Williams SM Brown RC , et al. HIIT in the real world: outcomes from a 12-month intervention in overweight adults. Med Sci Sports Exerc 2018; 50: 1818–1826. 29683919 10.1249/MSS.0000000000001642

[10] Hesketh K Jones H Kinnafick F , et al. Home-based HIIT and traditional MICT prescriptions improve cardiorespiratory fitness to a similar extent within an exercise referral scheme for at-risk individuals. Front Physiol 2021: 2015: 750283. 10.3389/fphys.2021.750283 PMC8631444 34858205

[11] Shore CB Hubbard G Gorely T , et al. Insufficient reporting of factors associated with exercise referral scheme uptake, attendance, and adherence: a systematic review of reviews. J Phys Act Health 2019; 16: 667–676. 31203705 10.1123/jpah.2018-0341

[12] Palazzo C Klinger E Dorner V , et al. Barriers to home-based exercise program adherence with chronic low back pain: patient expectations regarding new technologies. Ann Phys Rehabil Med 2016; 59: 107–113. 27050664 10.1016/j.rehab.2016.01.009

[13] Morgan F Battersby A Weightman AL , et al. Adherence to exercise referral schemes by participants – what do providers and commissioners need to know? A systematic review of barriers and facilitators. BMC Public Health 2016; 16: 27. 26944952 10.1186/s12889-016-2882-7 PMC4779205

[14] Bachmann C Oesch P Bachmann S . Recommendations for improving adherence to home-based exercise: a systematic review. Physikalische Medizin, Rehabilitationsmedizin, Kurortmedizin 2018; 28: 20–31.

[15] Jung M Locke S Bourne J , et al. Cardiorespiratory fitness and accelerometer-determined physical activity following one year of free-living high-intensity interval training and moderate-intensity continuous training: a randomized trial. Int J Behav Nutr Phys Act 2020; 17: 1–10. 32102667 10.1186/s12966-020-00933-8 PMC7045584

[16] Eldridge SM Chan CL Campbell MJ , et al. CONSORT 2010 statement: extension to randomised pilot and feasibility trials. Br Med J 2016; 355: i5239. 27777223 10.1136/bmj.i5239 PMC5076380

[17] Hoffmann TC Glasziou PP Boutron I , et al. Better reporting of interventions: template for intervention description and replication (TIDieR) checklist and guide. BMJ: Br Med J 2014; 348: g1687. 24609605 10.1136/bmj.g1687

[18] The UK Chief Medical Officers physical activity guidelines report. (cited 2019 Sept 9), https://www.gov.uk/government/publications/physical-activity-guidelines-uk-chief-medical-officers-report.

[19] Mendes R Sousa N Almeida A , et al. Exercise prescription for patients with type 2 diabetes—a synthesis of international recommendations: narrative review. Br J Sports Med 2016; 50: 1379–1381. 26719499 10.1136/bjsports-2015-094895

[20] Maxwell JD France M Finnigan LEM , et al. Can exercise training enhance the repeated remote ischaemic preconditioning stimulus on peripheral and cerebrovascular function in high-risk individuals? Eur J Appl Physiol 2021; 121: 1167–1178. 33507363 10.1007/s00421-020-04580-6 PMC7966185

[21] Scott SN Cocks M Andrews RC , et al. High-intensity interval training improves aerobic capacity without a detrimental decline in blood glucose in people with type 1 diabetes. J Clin Endocrinol Metab 2018; 104: 604–612. 10.1210/jc.2018-01309 30281094

[22] Borg G . Borg’s perceived exertion and pain scales. Champaign, IL: Human Kinetics, 1998.

[23] Hesketh K Low J Andrews R , et al. Mobile health biometrics to enhance exercise and physical activity adherence in type 2 diabetes (MOTIVATE-T2D): protocol for a feasibility randomised controlled trial. BMJ Open 2021; 11: e052563. 10.1136/bmjopen-2021-052563 PMC8628337 34836904

[24] American College of Sports Medicine. ACSM’s Guidelines for Exercise Testing and Prescription. 9th ed. Baltimore, MD: Wolters Kluwer; 2014.

[25] Bandura A . Social foundations of thought and action. Englewood Cliffs, NJ: Prentice-Hall, Inc., 1986.

[26] Michie S van Stralen MM West R . The behaviour change wheel: a new method for characterising and designing behaviour change interventions. Implement Sci 2011; 6: 42. 21513547 10.1186/1748-5908-6-42 PMC3096582

[27] Godin G Shephard R . Godin leisure-time exercise questionnaire. Med Sci Sports Exerc 1997; 29: S36–S38.

[28] Van Hees VT Fang Z Langford J , et al. Autocalibration of accelerometer data for free-living physical activity assessment using local gravity and temperature: an evaluation on four continents. J Appl Physiol 2014; 117: 738–744. 25103964 10.1152/japplphysiol.00421.2014 PMC4187052

[29] Van Hees VT Gorzelniak L Dean León EC , et al. Separating movement and gravity components in an acceleration signal and implications for the assessment of human daily physical activity. PloS one 2013; 8: e61691. 23626718 10.1371/journal.pone.0061691 PMC3634007

[30] Hildebrand M Van Hees VT Hansen BH , et al. Age group comparability of raw accelerometer output from wrist-and hip-worn monitors. Med Sci Sports Exercise 2014; 46: 1816–1824. 10.1249/MSS.0000000000000289 24887173

[31] Hildebrand M Hansen BH van Hees VT , et al. Evaluation of raw acceleration sedentary thresholds in children and adults. Scand J Med Sci Sports 2017; 27: 1814–1823. 27878845 10.1111/sms.12795

[32] Scott SN Shepherd SO Andrews RC , et al. A multidisciplinary evaluation of a virtually supervised home-based high-intensity interval training intervention in people with type 1 diabetes. Diabetes Care 2019; 42: 2330–2333. 31530660 10.2337/dc19-0871

[33] MINISTRY OF HOUSING COMMUNITIES & LOCAL GOVERNMENT. English Indices of Deprivation 2015 [Online]. Government Official Statistics, https://www.gov.uk/government/statistics/english-indices-of-deprivation-2015 (2015).

[34] Kushi LH Kaye SA Folsom AR , et al. Accuracy and reliability of self-measurement of body girths. Am J Epidemiol 1988; 128: 740–748. 3421240 10.1093/oxfordjournals.aje.a115027

[35] Brown R Randhawa A Canning K , et al. Waist circumference at five common measurement sites in normal weight and overweight adults: which site is most optimal? Clin Obes 2018; 8: 21–29. 29218787 10.1111/cob.12231

[36] Shimbo D Artinian NT Basile JN , et al. Self-measured blood pressure monitoring at home: a joint policy statement from the American Heart Association and American Medical Association. Circulation 2020; 142: e42–e63. 32567342 10.1161/CIR.0000000000000803

[37] Billingham SA Whitehead AL Julious SA . An audit of sample sizes for pilot and feasibility trials being undertaken in the United Kingdom registered in the United Kingdom Clinical Research Network database. BMC Med Res Methodol 2013; 13: 1–6. 23961782 10.1186/1471-2288-13-104 PMC3765378

[38] Sim J Lewis M . The size of a pilot study for a clinical trial should be calculated in relation to considerations of precision and efficiency. J Clin Epidemiol 2012; 65: 301–308. 22169081 10.1016/j.jclinepi.2011.07.011

[39] Cohen MP . Determining sample sizes for surveys with data analyzed by hierarchical linear models. J Off Stat 1998; 14: 267.

[40] Landis JR Koch GG . The measurement of observer agreement for categorical data. Biometrics 1977; 33: 159–174. 843571

[41] Bland JM Altman D . Statistical methods for assessing agreement between two methods of clinical measurement. The Lancet 1986; 327: 307–310. 2868172

[42] Braun V Clarke V . Using thematic analysis in psychology. Qual Res Psychol 2006; 3: 77–101.

[43] Braun V Clarke V . Thematic analysis. In: Cooper H, Camic PM, Long DL, et al. (eds) APA Handbook of research methods in psychology, vol 2: research designs: quantitative, qualitative, neuropsychological, and biological. Washington, DC, US: American Psychological Association, 2012, pp.57–71.

[44] Clarke V Braun V Hayfield N . Thematic analysis. Qualitative Psychology: A Practical Guide to Research Methods 2015; 222: 248.

[45] Ritchie J Spencer L . Qualitative data analysis for applied policy research. The Qualitative Researcher’s Companion 2002; 573: 305–329.

[46] Taylor AH Doust J Webborn N . Randomised controlled trial to examine the effects of a GP exercise referral programme in Hailsham, East Sussex, on modifiable coronary heart disease risk factors. J Epidemiol Community Health 1998; 52: 595–601. 10320861 10.1136/jech.52.9.595 PMC1756762

[47] Baskerville R Ricci-Cabello I Roberts N , et al. Impact of accelerometer and pedometer use on physical activity and glycaemic control in people with type 2 diabetes: a systematic review and meta-analysis. Diabetic Med 2017; 34: 612–620. 28173623 10.1111/dme.13331

[48] Compernolle S DeSmet A Poppe L , et al. Effectiveness of interventions using self-monitoring to reduce sedentary behavior in adults: a systematic review and meta-analysis. Int J Behav Nutr Phys Act 2019; 16: 1–16. 31409357 10.1186/s12966-019-0824-3 PMC6693254

[49] Morgan F Battersby A Weightman AL , et al. Adherence to exercise referral schemes by participants–what do providers and commissioners need to know? A systematic review of barriers and facilitators. BMC Public Health 2016; 16: 1–11. 26944952 10.1186/s12889-016-2882-7 PMC4779205

[50] Bandura A . The nature and structure of self-efficacy. In: Self-efficacy: the exercise of control. New York, NY: WH Freeman and Company, 1997, pp.37–78.

[51] Miller FL O’Connor DP Herring MP , et al. Exercise dose, exercise adherence, and associated health outcomes in the TIGER study. Med Sci Sports Exercise 2014; 46: 69–75. 10.1249/MSS.0b013e3182a038b9 PMC3867583 23793231

[52] Kinnafick F-E Thøgersen-Ntoumani C Shepherd SO , et al. In it together: a qualitative evaluation of participant experiences of a 10-week, group-based, workplace HIIT program for insufficiently active adults. J Sport Exerc Psychol 2018; 40: 10–19. 29521569 10.1123/jsep.2017-0306

[53] Nes BM Gutvik CR Lavie CJ , et al. Personalized activity intelligence (PAI) for prevention of cardiovascular disease and promotion of physical activity. Am J Med 2017; 130: 328–336. 27984009 10.1016/j.amjmed.2016.09.031

[54] Taylor A Taylor RS Ingram W , et al. Randomised controlled trial of an augmented exercise referral scheme using web-based behavioural support for inactive adults with chronic health conditions: the e-coachER trial. Br J Sports Med 2021; 55: 444–450. 33247001 10.1136/bjsports-2020-103121 PMC8020080

[55] Matthew CE . Calibration of accelerometer output for adults. Med Sci Sports Exercise 2005; 37: S512–S522. 10.1249/01.mss.0000185659.11982.3d 16294114

[56] Paravidino VB Mediano MFF Crochemore-Silva I , et al. The compensatory effect of exercise on physical activity and energy intake in young men with overweight: the EFECT randomised controlled trial. Physiol Behav 2021; 229: 113249. 33221391 10.1016/j.physbeh.2020.113249

[57] McDermott MM Newman AB . Remote research and clinical trial integrity during and after the coronavirus pandemic. Jama 2021; 325: 1935–1936. 33885728 10.1001/jama.2021.4609

[58] Noonan D Simmons LA . Navigating nonessential research trials during COVID19: the push we needed for using digital technology to increase access for rural participants? The Journal of rural health: official journal of the American Rural Health Association and the National Rural Health Care Association 2021; 37: 185. 32282959 10.1111/jrh.12446 PMC7262024

[59] Font JC Hernández-Quevedo C McGuire A . Persistence despite action? Measuring the patterns of health inequality in England (1997–2007). Health Policy 2011; 103: 149–159. 21816501 10.1016/j.healthpol.2011.07.002

[60] Young L Barnason S Do V . Review strategies to recruit and retain rural patient participating self-management behavioral trials. Online J Rural Res Policy 2015; 10: 1. 28580049 10.4148/1936-0487.1070 PMC5451124

[61] Unger JM Moseley A Symington B , et al. Geographic distribution and survival outcomes for rural patients with cancer treated in clinical trials. JAMA Network Open 2018; 1: e181235–e181235. 30646114 10.1001/jamanetworkopen.2018.1235 PMC6324281

[62] Eames M Ben-Shlomo Y Marmot MG . Social deprivation and premature mortality: regional comparison across England. Br Med J 1993; 307: 1097–1102. 8251806 10.1136/bmj.307.6912.1097 PMC1679128

[63] Lee D-c Artero EG Sui X , et al. Mortality trends in the general population: the importance of cardiorespiratory fitness. J Psychopharmacol 2010; 24: 27–35. 10.1177/1359786810382057 PMC2951585 20923918

[64] Jackson AS Blair SN Mahar MT , et al. Prediction of functional aerobic capacity without exercise testing. Med Sci Sports Exercise 1990; 22: 863–870. 10.1249/00005768-199012000-00021 2287267

[65] Bennett H Parfitt G Davison K , et al. Validity of submaximal step tests to estimate maximal oxygen uptake in healthy adults. Sports Med 2016; 46: 737–750. 26670455 10.1007/s40279-015-0445-1

[66] Klepin K Wing D Higgins M , et al. Validity of cardiorespiratory fitness measured with fitbit compared to v˙ O2max. Med Sci Sports Exercise 2019; 51: 2251. 10.1249/MSS.0000000000002041 PMC7028477 31107835

[67] Carrier B Creer A Williams LR , et al. Validation of garmin fenix 3 HR fitness tracker biomechanics and metabolics (VO2max). J Measurement Phys Behav 2020; 3: 331–337.

