# Supplementary material for: Adherence to unsupervised exercise in sedentary individuals: A randomised feasibility trial of two mobile health interventions
Source: Digit Health. 2023 Jun 28;9:20552076231183552. doi: 10.1177/20552076231183552 (PMC10328121; doi:10.1177/20552076231183552)
Supplement: sj-docx-1-dhj-10.1177_20552076231183552 - Supplemental material for Adherence to unsupervised exercise in sedentary individuals: A randomised feasibility trial of two mobile health interventions [file sj-docx-1-dhj-10.1177_20552076231183552.docx]

**Supplementary Files – Names & Descriptions**

**Supplementary Files**

Supplementary File 1. CONSORT extension for Pilot and Feasibility Trials Checklist

Supplementary File 2. Template for Intervention Description and Replication (TIDieR)

**Supplementary Figures**

Supplementary Figure 1. A percentage of participants still completing training sessions.

Supplementary Figure 2. Accelerometer (mg) and HR data (%HRmax) traces recorded throughout bouts of HIIT (A) and VIT (B) conducted on a cycle ergometer. Red line is indicative of the vigorous intensity threshold (>429mg/70%HRmax), green line is indicative of the moderate intensity threshold (>101mg/60%HRmax) (15).

**Supplementary Tables**

Supplementary Table 1. Exercise prescription for moderate-intensity continuous training

Supplementary Table 2. Exercise prescription for vigorous-intensity training

Supplementary Table 3. Exercise prescription for high-intensity interval training

Supplementary Table 4. Exercise prescription for resistance training

Supplementary Table 5. Details of the counselling intervention

Supplementary Table 6. Topic guide baseline interviews.

Supplementary Table 7. Topic guide post-intervention interviews for MOTIVATE participants.

Supplementary Table 8. Topic guide post-intervention interviews for online resources participants.

Supplementary Table 9. Compliance criteria for exercise types

Supplementary Table 10. Survey questions post-intervention for MOTIVATE participants.

Supplementary Table 11. Survey questions post-intervention for online resources participants.

Supplementary Table 12. Survey question following baseline measures.

Supplementary Table 13. Perceived facilitators and barriers to exercise between intervention groups

Supplementary Table 14. Responses from the baseline testing survey
