# Supplementary material for: Adherence to unsupervised exercise in sedentary individuals: A randomised feasibility trial of two mobile health interventions
Source: Digit Health. 2023 Jun 28;9:20552076231183552. doi: 10.1177/20552076231183552 (PMC10328121; doi:10.1177/20552076231183552)
Supplement: sj-docx-2-dhj-10.1177_20552076231183552 - Supplemental material for Adherence to unsupervised exercise in sedentary individuals: A randomised feasibility trial of two mobile health interventions [file sj-docx-2-dhj-10.1177_20552076231183552.docx]

Supplementary Table 1. Exercise prescription for moderate-intensity continuous training

| Week | Total Session Duration (min) | Warm-up: Workout Duration (min) | Intensity (%HR_max_) (Warm-up: Workout) |
| --- | --- | --- | --- |
| 1-2 | 25 | 5:20 | 50-60:  50-70 |
| 3-4 | 30 | 5:25 |  |
| 5-6 | 35 | 5:30 |  |
| 7-8 | 40 | 5:35 |  |
| 9-10 | 45 | 5:40 |  |
| 11-12 | 50 | 5:45 |  |
