# Supplementary material for: Adherence to unsupervised exercise in sedentary individuals: A randomised feasibility trial of two mobile health interventions
Source: Digit Health. 2023 Jun 28;9:20552076231183552. doi: 10.1177/20552076231183552 (PMC10328121; doi:10.1177/20552076231183552)
Supplement: sj-docx-3-dhj-10.1177_20552076231183552 - Supplemental material for Adherence to unsupervised exercise in sedentary individuals: A randomised feasibility trial of two mobile health interventions [file sj-docx-3-dhj-10.1177_20552076231183552.docx]

Supplementary Table 2. Exercise prescription for vigorous-intensity training

| Week | Total Session Duration (min) | Low:High Workout Duration (min) | Warm-up: High: Low Phase Duration (min) | Repetition of Phases | Intensity (%HR_Max_)  (Low:High) |
| --- | --- | --- | --- | --- | --- |
| 1 | 20 | 14:6 | 5:2:3 | 3 | 50-60: 60-70 |
| 2 | 20 | 14:6 | 5:3:2 | 3 | 50-60: 60-70 |
| 3 | 25 | 13:12 | 5:3:2 | 4 | 50-60: 65-75 |
| 4 | 25 | 13:12 | 5:4:1 | 4 | 50-60: 65-75 |
| 5 | 25 | 9:16 | 5:4:1 | 4 | 50-60: 70-80 |
| 6 | 25 | 9:16 | 5:4:1 | 4 | 50-60: 70-80 |
| 7 | 29 | 9:20 | 5:5:1 | 4 | 50-60: 70-80 |
| 8 | 32 | 8:24 | 5:8:1 | 3 | 50-60: 70-80 |
| 9 | 30 | 7:24 | 5:12:1 | 2 | 50-60: 70-80 |
| 10 | 30 | 7:24 | 5:12:1 | 2 | 50-60: 70-80 |
| 11 | 30 | 5:25 | 5:25:0 | 1 | 50-60: 70-80 |
| 12 | 30 | 5:25 | 5:25:0 | 1 | 50-60: 70-80 |
