# Supplementary material for: Adherence to unsupervised exercise in sedentary individuals: A randomised feasibility trial of two mobile health interventions
Source: Digit Health. 2023 Jun 28;9:20552076231183552. doi: 10.1177/20552076231183552 (PMC10328121; doi:10.1177/20552076231183552)
Supplement: sj-docx-4-dhj-10.1177_20552076231183552 - Supplemental material for Adherence to unsupervised exercise in sedentary individuals: A randomised feasibility trial of two mobile health interventions [file sj-docx-4-dhj-10.1177_20552076231183552.docx]

Supplementary Table 3. Exercise prescription for high-intensity interval training

| Week | Total Session Duration (min) | Warm-up: Workout Duration | No Intervals | Intensity (%HR_Max_) (Warm-up: Workout) |
| --- | --- | --- | --- | --- |
| 1-2 | 12 | 2:10 | 5 | 50-70: ≥80 |
| 3-4 | 14 | 2:12 | 6 |  |
| 5-6 | 16 | 2:14 | 7 |  |
| 7-8 | 18 | 2:16 | 8 |  |
| 9-10 | 20 | 2:18 | 9 |  |
| 11-12 | 22 | 2:20 | 10 |  |
