# Supplementary material for: Adherence to unsupervised exercise in sedentary individuals: A randomised feasibility trial of two mobile health interventions
Source: Digit Health. 2023 Jun 28;9:20552076231183552. doi: 10.1177/20552076231183552 (PMC10328121; doi:10.1177/20552076231183552)
Supplement: sj-docx-5-dhj-10.1177_20552076231183552 - Supplemental material for Adherence to unsupervised exercise in sedentary individuals: A randomised feasibility trial of two mobile health interventions [file sj-docx-5-dhj-10.1177_20552076231183552.docx]

Supplementary Table 4. Exercise prescription for resistance training

| Week | Total Session Duration (min) | Warm-up: Workout Duration | No. of exercises | No of Sets | Time per set (s) | Rest between exercises/sets (s) |
| --- | --- | --- | --- | --- | --- | --- |
| 1-2 | 26 | 2: 24 | 5 | 3 | 30 | 60/120 |
| 3-4 | 28 | 2: 26 |  |  | 40 | 60/120 |
| 5-6 | 28 | 2:26 |  |  | 50 | 45/120 |
| 7-8 | 39 | 2:37 | 6 | 4 | 40 | 45/120 |
| 9-10 | 38 | 2:36 |  |  | 50 | 30/120 |
| 11-12 | 42 | 2:40 |  |  | 60 | 30/120 |
