# Supplementary material for: Adherence to unsupervised exercise in sedentary individuals: A randomised feasibility trial of two mobile health interventions
Source: Digit Health. 2023 Jun 28;9:20552076231183552. doi: 10.1177/20552076231183552 (PMC10328121; doi:10.1177/20552076231183552)
Supplement: sj-docx-6-dhj-10.1177_20552076231183552 - Supplemental material for Adherence to unsupervised exercise in sedentary individuals: A randomised feasibility trial of two mobile health interventions [file sj-docx-6-dhj-10.1177_20552076231183552.docx]

Supplementary Table 5. Details of the counselling intervention

|  | Date | Details |
| --- | --- | --- |
| Consultation 1 | Prior to intervention | Initial meeting to assess current beliefs/concerns, explore the benefits of exercise and agree on a SMART (specific, measurable, achievable, relevant and time-bound) plan |
| Consultation 2 | Prior to intervention | Development of the personal exercise program and education on how to use the mHealth technology |
| Consultation 3 | 4 weeks | Patient feedback and refinement of the exercise program with the aim of progressing the program. Data recorded in the smart phone app and online coaching platform was used to guide discussions |
| Consultation 4 | 12 weeks | Patient feedback and review of progress. Discussion on strategies for maintaining exercise and PA. Data recorded in the smart phone app and online coaching platform was used to guide discussions |
