# Supplementary material for: Adherence to unsupervised exercise in sedentary individuals: A randomised feasibility trial of two mobile health interventions
Source: Digit Health. 2023 Jun 28;9:20552076231183552. doi: 10.1177/20552076231183552 (PMC10328121; doi:10.1177/20552076231183552)
Supplement: sj-docx-7-dhj-10.1177_20552076231183552 - Supplemental material for Adherence to unsupervised exercise in sedentary individuals: A randomised feasibility trial of two mobile health interventions [file sj-docx-7-dhj-10.1177_20552076231183552.docx]

Supplementary Table 6. Topic guide baseline interviews.

| Key questions | Probes |
| --- | --- |
| How did you find the resources and training provided before the home testing? | - - How did it help (videos, booklets, checklist, phone call with researcher) |
|  | - - Would you have found it useful for the researcher to be with you during the testing, via video or phone? |
|  | - - Recommendations for improvement |
| Did you have any issues receiving the testing package? |  |
| What did you think about the measures you collected during the scheme? | - - Thoughts on specific outcome measures (questionnaires/bloods/blood pressure etc) |
|  | - - Understanding; interest; comfort; difficulty |
|  | - - Number of measures |
|  | - - Do you think we measured important outcomes? |
| What did you think about the 7-day period where you wore the PA monitor and flash glucose monitor (explain FGM if needed)? | - - Activity monitor |
|  | - - Understanding; interest; comfort; difficulty |
|  | - - Number of measures |
|  | - - Do you think we measured what was important? Did we miss anything? |
|  | - - Was it easy to return the monitors to the research team? |
|  | MOTIVATE only:   - - Was it easy to download and use the mobile Apps (Zoom and Polar Flow)? |
|  | MOTIVATE only:   - - Was it easy to pair the devices (Polar Flow, Ignite, OH1)? |
