# Supplementary material for: Adherence to unsupervised exercise in sedentary individuals: A randomised feasibility trial of two mobile health interventions
Source: Digit Health. 2023 Jun 28;9:20552076231183552. doi: 10.1177/20552076231183552 (PMC10328121; doi:10.1177/20552076231183552)
Supplement: sj-docx-8-dhj-10.1177_20552076231183552 - Supplemental material for Adherence to unsupervised exercise in sedentary individuals: A randomised feasibility trial of two mobile health interventions [file sj-docx-8-dhj-10.1177_20552076231183552.docx]

Supplementary Table 7. Topic guide post-intervention interviews for MOTIVATE participants.

| Key questions | Probes |
| --- | --- |
| Can you talk me through your experience of the exercise programme? | - - types of exercise; difficulty; progression; engaging; choice |
|  | - - Anything you didn’t like / found difficult |
|  | - - Recommendations for improvement |
| How well supported did you feel in terms of the exercise advice? | - - Did you feel more or less supported by joining the study? |
|  | - - Who supported you? |
|  | - - Do you think this helped your exercise behaviour? |
| Is there any additional support that could have further encourage you to exercise? |  |
| Do you feel more or less confident about taking part in exercise following the intervention? |  |
| Did you find any factors particularly helpful in increasing your exercise levels? |  |
| Did you face any barriers to increasing your exercise levels? | - - Did you overcome these: if so, how? |
| How useful do you feel the exercise counselling sessions were for supporting a change in your exercise habits? | - - What were the pro and cons of talking to your exercise specialist? |
|  | - - Would sessions have been better face-to-face? |
|  | - - Session duration |
|  | - - Number/ frequency of sessions |
| Did the text messages from your exercise specialist encourage you to keep exercising? | - - Could the language in text have been improved? |
|  | - - Number/ frequency of texts |
|  | - - Did you feel able to reply to the messages? |
| How useful did you find the mobile App (Polar Flow) for supporting a change in your exercise habits? | - - How useful did you find the activity target? |
|  | - - Did you encounter any problems with the App? |
|  | - - How user friendly was the App? |
|  | - - How often did you look at the app? |
| How useful did you find the fitness watch (Polar Flow) for supporting a change in your exercise habits? | - - Was the watch easy to use (difficulties syncing the watch and App)? |
|  | - - Did you like the design of the watch (bulky, big enough screen)? |
|  | - - Did the pre-set exercise sessions help you? |
|  | - - Would you change the watch face in any way? |
|  | - - Did you find HR feedback useful? |
| Do you think the MOTIVATE LJMU website supported a change in your exercise habits? | - - Elements of the website that worked well |
|  | - - Elements of the website that could be improved |
| Which additional support element (App, texts, watch, website) did you find most useful for supporting a change in your exercise habits? |  |
| Where did you first hear about the trial from? Who approached you? |  |
| What appealed to you about taking part in the study in the first place? What were your reasons? |  |
| Was there anything that put you off taking part in the study? |  |
| Did the study meet your expectations from the advertisements, PIS and initial meeting? |  |
| How easy was it to complete the measures at home? |  |
| Did you feel well supported taking measures at home? |  |
| How easy to complete and understand were the questionnaires you received? |  |
| Was communication with the research team well managed? |  |
| If there was one thing we could do to make the study easier for you to take part in, what would it be? |  |
| We are planning to undertake a larger scale study similar to the one you have taken part in. What sort of things would you change about the study that might make people want to take part in it more? |  |
| Overall, what did you feel was good or bad about taking part in the study? |  |
