# Supplementary material for: Adherence to unsupervised exercise in sedentary individuals: A randomised feasibility trial of two mobile health interventions
Source: Digit Health. 2023 Jun 28;9:20552076231183552. doi: 10.1177/20552076231183552 (PMC10328121; doi:10.1177/20552076231183552)
Supplement: sj-docx-9-dhj-10.1177_20552076231183552 - Supplemental material for Adherence to unsupervised exercise in sedentary individuals: A randomised feasibility trial of two mobile health interventions [file sj-docx-9-dhj-10.1177_20552076231183552.docx]

Supplementary Table 8. Topic guide post-intervention interviews for online resources participants.

| Key questions | Probes |
| --- | --- |
| Can you talk me through your experience of the exercise programme? | - - types of exercise; difficulty; progression; engaging; choice |
|  | - - Anything you didn’t like / found difficult |
|  | - - Recommendations for improvement |
| How well supported did you feel in terms of the exercise advice? | - - Did you feel more or less supported by joining the study? |
|  | - - Who supported you? |
|  | - - Do you think this helped your exercise behaviour? |
| Is there any additional support that could have further encourage you to exercise? |  |
| Do you feel more or less confident about taking part in exercise following the intervention? |  |
| Did you find any factors particularly helpful in increasing your exercise levels? |  |
| Did you face any barriers to increasing your exercise levels? | - - Did you overcome these: if so how? |
| Do you think the MOTIVATE LJMU website supported a change in your exercise habits? | - - Elements of the website that worked well |
|  | - - Elements of the website that could be improved |
| Did you like the OH1? | - - What didn’t you like about it? |
|  | - - Did you forget to wear the OH1? |
|  | - - Did you ever forget to start and stop the session? |
|  | - - Would you use the OH1 in the same way (to gather more data) after the study? |
| Where did you first hear about the trial from? Who approached you? |  |
| What appealed to you about taking part in the study in the first place? What were your reasons? |  |
| Was there anything that put you off taking part in the study? |  |
| Did the study meet your expectations from the advertisements, PIS and initial meeting? |  |
| How easy was it to complete the measures at home? |  |
| Did you feel well supported taking measures at home? |  |
| How easy to complete and understand were the questionnaires you received? |  |
| Was communication with the research team well managed? |  |
| If there was one thing we could do to make the study easier for you to take part in what would it be? |  |
| We are planning to undertake a larger scale study similar to the one you have taken part in. What sort of things would you change about the study that might make people want to take part in it more? |  |
| Overall, what did you feel was good or bad about taking part in the study? |  |
