# Supplementary material for: Adherence to unsupervised exercise in sedentary individuals: A randomised feasibility trial of two mobile health interventions
Source: Digit Health. 2023 Jun 28;9:20552076231183552. doi: 10.1177/20552076231183552 (PMC10328121; doi:10.1177/20552076231183552)
Supplement: sj-docx-10-dhj-10.1177_20552076231183552 - Supplemental material for Adherence to unsupervised exercise in sedentary individuals: A randomised feasibility trial of two mobile health interventions [file sj-docx-10-dhj-10.1177_20552076231183552.docx]

Supplementary Table 9. Compliance criteria for exercise types

|  | Compliance criteria | |
| --- | --- | --- |
| Exercise type | Duration | Intensity |
| MICT | ≥ what was prescribed | Mean session HR ≥ 60% HR_max_ |
| VIT | ≥ what was prescribed | Mean session HR ≥ the predicted mean HR (based on the prescription, see Supplementary Table 6) |
| HIIT | Completed the prescribed number of intervals | Achieving a HR ≥80% HR_max_ during the session |
| RT | ≥ what was prescribed | *N/A* |

MICT; moderate-intensity continuous training, VIT; vigorous-intensity training, HIIT; high-intensity interval training, RT; resistance training, HR; heart rate, HRmax; heart rate maximum predicted using 220-age.
