# Supplementary material for: Adherence to unsupervised exercise in sedentary individuals: A randomised feasibility trial of two mobile health interventions
Source: Digit Health. 2023 Jun 28;9:20552076231183552. doi: 10.1177/20552076231183552 (PMC10328121; doi:10.1177/20552076231183552)
Supplement: sj-docx-11-dhj-10.1177_20552076231183552 - Supplemental material for Adherence to unsupervised exercise in sedentary individuals: A randomised feasibility trial of two mobile health interventions [file sj-docx-11-dhj-10.1177_20552076231183552.docx]

Supplementary Table 10. Survey questions post-intervention for MOTIVATE participants.

|  | Question |
| --- | --- |
| 1 | Did you find any factors particularly helpful in increasing your exercise levels. Please provide up to three facilitators |
| 2 | What barriers did you face to increasing your exercise levels. Please provide up to three barriers |
| 3 | What did you like about the extra support (e.g. Polar Flow app, fitness watch, counselling sessions and feedback from researcher and HR feedback during exercise)? What could be improved? |
| 4 | What did you like about the counselling sessions? What could be improved |
| 5 | What did you like about the text messages? What could be improved |
| 6 | Were there any challenges to accessing or using the participant app? How could these challenges be overcome? |
| 7 | Were there any challenges to using the fitness watch? How could these challenges be overcome? |
| 8 | Were there any challenges to using the website? How do you think these challenges could be overcome? |
