# Supplementary material for: Adherence to unsupervised exercise in sedentary individuals: A randomised feasibility trial of two mobile health interventions
Source: Digit Health. 2023 Jun 28;9:20552076231183552. doi: 10.1177/20552076231183552 (PMC10328121; doi:10.1177/20552076231183552)
Supplement: sj-docx-13-dhj-10.1177_20552076231183552 - Supplemental material for Adherence to unsupervised exercise in sedentary individuals: A randomised feasibility trial of two mobile health interventions [file sj-docx-13-dhj-10.1177_20552076231183552.docx]

Supplementary Table 12. Survey question following baseline measures.

| 1. Did you have any issues receiving the testing equipment parcel? | | | | | |
| --- | --- | --- | --- | --- | --- |
| Yes | No |  | | | |
| 2. For each of the procedures how easy did you find taking the measure at home? | | | | | |
|  | Extremely easy | Very easy | Somewhat easy | Slightly easy | Not at all easy |
| Measuring your height |  |  |  |  |  |
| Measuring your weight |  |  |  |  |  |
| Measuring your waist circumference |  |  |  |  |  |
| Taking your blood pressure |  |  |  |  |  |
| 3. How easy did you find completing the questionnaires online | | | | | |
|  | Extremely easy | Very easy | Somewhat easy | Slightly easy | Not at all easy |
| 4. Was it easy to post these devices back to the research team | | | | | |
|  | Extremely easy | Very easy | Somewhat easy | Slightly easy | Not at all easy |
