# Supplementary material for: Adherence to unsupervised exercise in sedentary individuals: A randomised feasibility trial of two mobile health interventions
Source: Digit Health. 2023 Jun 28;9:20552076231183552. doi: 10.1177/20552076231183552 (PMC10328121; doi:10.1177/20552076231183552)
Supplement: sj-docx-14-dhj-10.1177_20552076231183552 - Supplemental material for Adherence to unsupervised exercise in sedentary individuals: A randomised feasibility trial of two mobile health interventions [file sj-docx-14-dhj-10.1177_20552076231183552.docx]

Supplementary Table 13. Perceived facilitators and barriers to exercise between intervention groups

Numbers indicate the number of participants that gave a response related to each theme. Representative participant quotes are placed below each theme and sub-theme.

|  |  | MOTIVATE | | Online Resources | |
| --- | --- | --- | --- | --- | --- |
| Theme | Subtheme | Positive Responses | Negative Responses | Positive Responses | Negative Responses |
| Motivation | Accountability | Being Monitored by the team (13), being part of the study (2), health Check (1) |  | Being part of the study (4), health Check (2) |  |
|  |  | “*Having someone monitoring it without being present”* |  | *“A sense of commitment by being part of the programme.”* |  |
|  | Communication and feedback | Text messages (17), regular guidance (28), counselling sessions (1) |  |  |  |
|  |  | *“Text messages checking in on me and commenting on my sessions helped motivate me”* |  |  |  |
|  | Tracking | Using app to track (16), real-Time Heart rate Feedback (19) |  | Wearing a monitor (7) |  |
|  |  | *“The polar flow app and fitness watch was really useful in monitoring my activity and pushing me to ensure I hit my daily goal.”* |  | *“Wearing the Polar [OH1] heart rate monitor when exercising”* |  |
|  | Flexibility of the Intervention | Programme to follow (13), flexible programme (4), progressive programme (5), personalised (7), resources (2) | Work/life balance (15), lack of time (7), lack of motivation (2), tiredness (1) | Programme to follow (8), resources (8) | Work/life balance (10), lack of time (9), lack of Motivation (2), tiredness (8) |
|  |  | *“I liked the fact that it was tailored toward myself and what I wanted to achieve and complete”* | *“Long workdays made me tired, and I didn’t want to go out.”* | *“Having access to online videos to guide me through exercise”* | *“Sometimes difficult to find time in a busy workday to exercise.”* |
|  | Environmental | Social support (2) | Health (12), weather (5), social Support (1) | Weather (3), social support (1) | Health (13), weather (8), |
|  |  | *“Other people I knew were on the programme”* | *“Bad weather makes me not want to go outside”* | *“Planning time for it when good weather “* | *“Suffered with various injuries and not resting long enough to recover”* |
| Covid |  | Health Concerns as a result of COVID (1), change in schedule (1) | Unable to access facilities (4), isolating (3), ill health due to COVID (1) | Health Concerns as a result of COVID (3), change in schedule (2) | Unable to access facilities (1), isolating (3), ill health due to COVID (2) |
|  |  | *“Working from home”* | *“Having to self-isolate”* | *“The potential impact of Covid on overweight people like myself “* | *“Being classed as vulnerable and advised not to go to the gym owing to Covid”* |
