# Supplementary material for: Adherence to unsupervised exercise in sedentary individuals: A randomised feasibility trial of two mobile health interventions
Source: Digit Health. 2023 Jun 28;9:20552076231183552. doi: 10.1177/20552076231183552 (PMC10328121; doi:10.1177/20552076231183552)
Supplement: sj-docx-15-dhj-10.1177_20552076231183552 - Supplemental material for Adherence to unsupervised exercise in sedentary individuals: A randomised feasibility trial of two mobile health interventions [file sj-docx-15-dhj-10.1177_20552076231183552.docx]

Supplementary Table 14. Responses from the baseline testing survey

|  | Yes  n (%) | No  n (%) |  |  |  |
| --- | --- | --- | --- | --- | --- |
| Did you have any issues receiving the testing equipment parcel? | 8 (9.6) | 75 (90.4) |  |  |  |
|  | Extremely easy  n (%) | Very easy  n (%) | Somewhat easy  n (%) | Slightly easy  n (%) | Not at all easy  n (%) |
| For each of the procedures how easy did you find taking the measure at home? |  |  |  |  |  |
| Height | 40 (48.2) | 35 (42.2) | 6 (7.2) | 0 (0) | 2 (2.4) |
| Weight | 68 (84) | 13 (16) | 0 (0) | 0 (0) | 0 (0) |
| Waist circumference | 47 (56.6) | 27 (32.5) | 7 (8.4) | 1 (1.2) | 1 (1.2) |
| Blood pressure | 39 (47.6) | 29 (35.4) | 12 (14.6) | 2 (2.4) | 0 (0) |
| How easy did you find completing the questionnaires online? | 59 (71.1) | 23 (27.7) | 1 (1.2) | 0 (0) | 0 (0) |
| Was it easy to post these devices back to the research team? | 58 (75.3) | 14 (18.2) | 3 (3.9) | 2 (2.6) | (0) |
